# A curated benchmark of enhancer-gene interactions for evaluating enhancer-target gene prediction methods

**DOI:** 10.1186/s13059-019-1924-8

**Published:** 2020-01-22

**Authors:** Jill E. Moore, Henry E. Pratt, Michael J. Purcaro, Zhiping Weng

**Affiliations:** Program in Bioinformatics and Integrative Biology, University of Massachusetts Medical School, Worcester, MA 01605 USA

**Keywords:** Enhancer, Transcriptional regulation, Target gene, Benchmark, Machine learning, Genomic interactions

## Abstract

**Background:**

Many genome-wide collections of candidate cis-regulatory elements (cCREs) have been defined using genomic and epigenomic data, but it remains a major challenge to connect these elements to their target genes.

**Results:**

To facilitate the development of computational methods for predicting target genes, we develop a Benchmark of candidate Enhancer-Gene Interactions (BENGI) by integrating the recently developed Registry of cCREs with experimentally derived genomic interactions. We use BENGI to test several published computational methods for linking enhancers with genes, including signal correlation and the TargetFinder and PEP supervised learning methods. We find that while TargetFinder is the best-performing method, it is only modestly better than a baseline distance method for most benchmark datasets when trained and tested with the same cell type and that TargetFinder often does not outperform the distance method when applied across cell types.

**Conclusions:**

Our results suggest that current computational methods need to be improved and that BENGI presents a useful framework for method development and testing.

**Electronic supplementary material:**

The online version of this article (10.1186/s13059-019-1924-8) contains supplementary material, which is available to authorized users.

## Background

With the rapid increases in genomic and epigenomic data in recent years, our ability to annotate regulatory elements across the human genome and predict their activities in specific cell and tissue types has substantially improved. Widely used approaches integrate multiple epigenetic signals such as chromatin accessibility, histone marks, and transcribed RNAs [1–7] to define collections of regulatory elements that can be used to study regulatory programs in diverse cell types and dissect the genetic variations associated with human diseases [5, 8–11].

To maximize the utility of regulatory elements, one must know which genes they regulate. We recently developed the Registry of candidate cis-Regulatory elements (cCREs), a collection of candidate regulatory genomic regions in humans and mice, by integrating chromatin accessibility (DNase-seq) data and histone mark ChIP-seq data from hundreds of biosamples generated by the ENCODE Consortium (http://screen.encodeproject.org). Over 75% of these cCREs have enhancer-like signatures (high chromatin accessibility as measured by a high DNase-seq signal and a high level of the enhancer-specific histone mark H3K27ac) and are located distal (> 2 kb) to an annotated transcription start site (TSS). For cCREs proximal to a TSS, it may be safe to assume that the TSS corresponds to the target gene, but to annotate the biological function of the TSS-distal cCREs and interpret the genetic variants that they harbor, we need to determine which genes they regulate.

Assigning enhancers to target genes on a genome-wide scale remains a difficult task. While one could assign an enhancer to the closest gene using linear distance, there are many examples of enhancers skipping over nearby genes in favor of more distal targets [12]. Experimental assays such as Hi-C and ChIA-PET survey physical interactions between genomic regions [13–17], and by overlapping the anchors of these interactions with annotated enhancers and promoters, we can infer regulatory connections. Approaches based on quantitative trait loci (QTL) associate genetic variants in intergenic regions with genes via the variation in their expression levels across multiple individuals in a human population [18, 19]. Recently, a single-cell perturbation approach extended this idea [20]. However, these assays are expensive to perform and have only been conducted at a high resolution in a small number of cell types. Therefore, we need to rely on computational methods to broadly predict enhancer-gene interactions.

One popular computational method for identifying enhancer-gene interactions is to correlate genomic and epigenomic signals at enhancers and gene promoters across multiple biosamples. This method is based on the assumption that enhancers and genes tend to be active or inactive in the same cell types. The first study to utilize this method linked enhancers with genes by correlating active histone mark signals at enhancers with gene expression across nine cell types [1]. Several groups subsequently used similar approaches to link enhancers and genes by correlating various combinations of DNase, histone mark, transcription factor, and gene expression data [8, 21–23]. While these methods successfully identified a subset of biologically relevant interactions, their performance has yet to be systematically evaluated.

Other groups have developed supervised machine-learning methods that train statistical models on sets of known enhancer-gene pairs. Most of these models use epigenomic signals (e.g., histone marks, TFs, DNase) at enhancers, promoters, or intervening windows as input features [24–27]. PEP-motif, on the other hand, uses sequence-based features [28]. The performance of these methods has not been systematically evaluated for several reasons. First, different methods use different definitions for enhancers ranging from EP300 peaks [26] to chromatin segments [27]. Second, these methods use different datasets to define their gold standards, such as ChIA-PET interactions [24, 26] or Hi-C loops [26, 27], along with different methods for generating negative pairs. Finally, many of these methods use a traditional randomized cross-validation scheme, which results in severe overfitting of some supervised models due to overlapping features [29, 30].

To facilitate the development of target gene prediction methods, we developed a collection of benchmark datasets by integrating the Registry of cCREs with experimentally derived genomic interactions. We then tested several published methods for linking enhancers with genes, including signal correlation and the supervised learning methods TargetFinder and PEP [27, 28]. Overall, we found that while TargetFinder was the best-performing method, it was only modestly better than a baseline distance method for most benchmark datasets when trained and tested on the same cell type, and Target Finder often did not outperform the distance method when applied across cell types. Our results suggest that current computational methods need to be improved and that our benchmark presents a useful framework for method development and testing.

## Results

### A Benchmark of candidate Enhancer-Gene Interactions (BENGI)

**Fig. 1:**
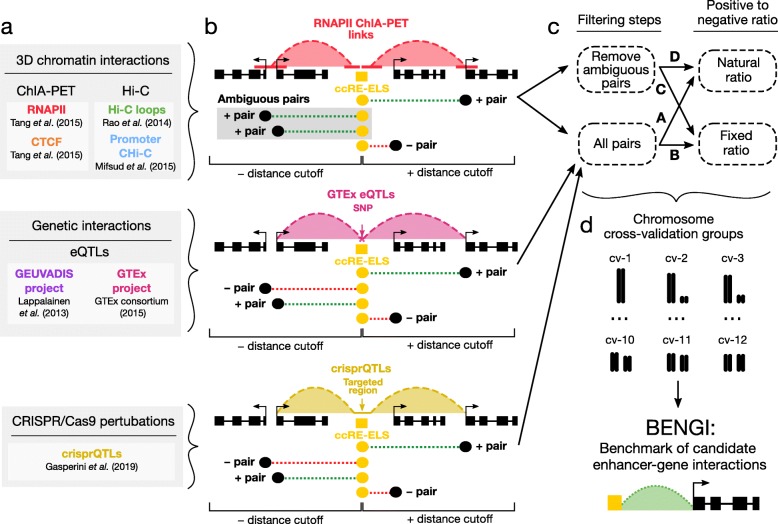
A benchmark of candidate enhancer-gene interactions (BENGI). **a** Experimental datasets used to curate BENGI interactions categorized by 3D chromatin interactions, genetic interactions, and CRISPR/Cas9 perturbations. **b** Methods of generating cCRE-gene pairs (dashed straight lines in green, shaded green, or red) from experimentally determined interactions or perturbation links (dashed, shaded arcs in red, pink, or gold). Each cCRE-gene pair derived from 3D chromatin interactions (top panel) has a cCRE-ELS (yellow box) intersecting one anchor of a link, and the pair is classified depending on the other anchor of the link: for a positive pair (dashed green line), the other anchor overlaps one or more TSSs of just one gene; for an ambiguous pair (dashed line with gray shading), the other anchor overlaps the TSSs of multiple genes; for a negative pair (dashed red line), the other anchor does not overlap with a TSS. Each cCRE-gene pair derived from genetic interactions or perturbation links (middle and bottom panels) has a cCRE-ELS (yellow box) intersecting an eQTL SNP or a CRISPR-targeted region, and the pair is classified as positive (dashed green line) if the gene is an eQTL or crisprQTL gene, while all the pairs that this cCRE forms with non-eQTL genes that have a TSS within the distance cutoff are considered negative pairs (dashed red line). **c** To reduce potential false positives obtained from 3D interaction data, we implemented a filtering step to remove ambiguous pairs (gray box in **b**) that link cCREs-ELS to more than one gene. This filtering step was not required for assays that explicitly listed the linked gene (eQTLs and crisprQTLs). Additionally, for comparisons between BENGI datasets, we also curated matching sets of interactions with a fixed positive-to-negative ratio. Therefore, a total of four BENGI datasets were curated for each 3D chromatin experiment (A, B, C, D), and two were curated for each genetic interaction and CRISPR/Cas-9 perturbation experiment (A, B). **d** To avoid overfitting of machine-learning algorithms, all cCRE-gene pairs were assigned to cross-validation (CV) groups based on their chromosomal locations. Positive and negative pairs on the same chromosome were assigned to the same CV group, and chromosomes with complementary sizes were assigned to the same CV group so that the groups contained approximately the same number of pairs

To effectively evaluate target gene prediction methods, we curated a Benchmark of candidate Enhancer-Gene Interactions (BENGI) by integrating our predicted enhancers, cCREs with enhancer-like signatures (cCREs-ELS), with 3D chromatin interactions, genetic interactions, and CRISPR/dCAS9 perturbations in a total of 21 datasets across 13 biosamples (Fig. 1a, Additional file 1: Tables S1 and Additional file 2: Table S2a). For 3D chromatin interactions, which include ChIA-PET, Hi-C, and CHi-C interactions, we selected all links with one anchor overlapping a distal cCRE-ELS and the other anchor falling within 2 kb of a GENCODE-annotated TSS (Fig. 1b, see “Methods”). For approximately three quarters of the total interactions, the anchor of the 3D chromatin interaction overlaps the proximal region of more than one gene, making the assignment of the exact gene target ambiguous. To assess the impact of these potentially ambiguous assignments, we created two versions of each 3D interaction benchmark dataset. In the first, we retained all cCRE-gene links; in the second, we removed links with ends within 2 kb of the TSSs of multiple genes (i.e., ambiguous pairs). For genetic interactions (cis-eQTLs) and CRISPR/dCas9 perturbations (crisprQTLs), we paired a cCRE-ELS with a gene if the cCRE overlapped the reported SNP or targeted region (Fig. 1b). In total, we curated over 162,000 unique cCRE-gene pairs across the 13 biosamples. Because these experimental datasets capture different aspects of enhancer-gene interactions (see statistical analyses in the next section), we retained the cCRE-gene pairs as separate datasets in BENGI.

To complement the positive cCRE-gene pairs in each BENGI dataset, we generated negative pairs for each cCRE-ELS by selecting all unpaired genes whose TSS was located within (either upstream or downstream) the 95th percentile distance from all positive cCRE-gene pairs in the dataset (Additional file 2: Table S2a, see “Methods”). These distance cutoffs ranged from 120 kb (RNAPII ChIA-PET in HeLa) to 1.83 Mb (Hi-C in K562). The percentages of positive pairs also varied from 1.8% (Hi-C in K562) to 23.5% (CHi-C in GM12878), and datasets with greater class imbalance (i.e., a smaller percentage of positive pairs) are inherently more challenging for a computational algorithm. To enable the comparison of algorithm performance across datasets, we further created datasets with a fixed ratio of one positive to four negatives for each BENGI dataset by randomly discarding the excess negatives. This strategy, along with the previously mentioned removal of ambiguous 3D chromatin interaction pairs, resulted in four BENGI datasets per ChIA-PET, Hi-C, or CHi-C experiment and two BENGI datasets per eQTL or crisprQTL experiment (Fig. 1c, Additional file 2: Table S2a). All pairs with a natural positive-negative ratio were used in our analyses unless otherwise noted.

To facilitate the training and testing of supervised machine-learning algorithms, we then assigned both positive and negative pairs to 12 cross-validation (CV) groups by chromosome such that pairs within the same chromosome were always assigned to the same CV group, while similar sizes were maintained for different CV groups by pairing one large chromosome with one small chromosome (chromCV, see “Methods”, Fig. 1d). Because GM12878 and other lymphoblastoid cell lines (LCLs) had the most BENGI datasets and have been extensively surveyed by the ENCODE and 1000 Genomes Consortia, we will highlight our analyses on the BENGI datasets from LCLs.

### Summary statistics of BENGI datasets

**Fig. 2:**
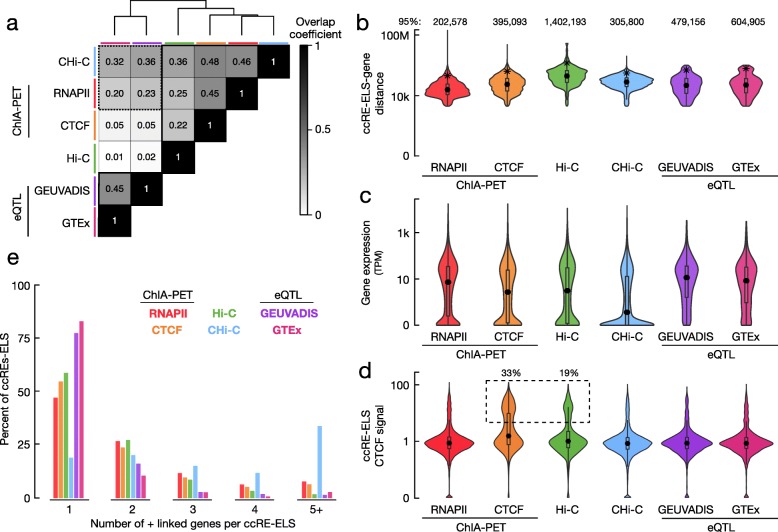
Characteristics of BENGI datasets. Six datasets in GM12878 or other LCLs were evaluated: RNAPII ChIA-PET (red), CTCF ChIA-PET (orange), Hi-C (green), CHi-C (blue), GEUVADIS eQTLs (purple), and GTEx eQTLs (pink), and the same color scheme is used for all panels. **a** Heatmap depicting the overlap coefficients between positive cCRE-gene pairs in each BENGI dataset. The datasets were clustered using the hclust algorithm, and the clustered datasets are outlined in black. **b** Violin plots depicting the distance distributions of positive cCRE-gene pairs for each BENGI dataset. The 95th percentile of each distribution is indicated by a star and presented above each plot. **c** Violin plots depicting the expression levels of genes in positive cCRE-gene pairs (in transcripts per million, TPM). **d** Violin plots depicting CTCF signal levels at cCREs-ELSs in positive cCRE-gene pairs. A dashed box indicates cCREs-ELS with a signal > 5. **e** Distributions of the number of genes positively linked with a cCRE-ELS across datasets

We asked whether the various types of chromatin, genetic, and CRISPR experiments might capture different types of enhancer-gene interactions. To answer this question, we carried out several statistical analyses across the BENGI datasets. First, we performed hierarchical clustering of the six BENGI datasets in GM12878/LCLs by the overlap coefficient—the number of positive cCRE-gene pairs shared between two datasets divided by the number of positives in the smaller dataset. We obtained two clusters: one comprising the two eQTL datasets and the other comprising the four chromatin interaction datasets (Fig. 2a). This overall grouping of the datasets was consistent with the characteristics of the experimental techniques (Table 1). Beyond the overall grouping, the two eQTL datasets exhibited higher overlap coefficients with the RNAPII ChIA-PET and CHi-C datasets (0.20–0.36) than with the Hi-C and CTCF ChIA-PET datasets (0.01–0.05). This reflects the promoter emphasis of the first four techniques, enriching for promoter-proximal interactions. In contrast, Hi-C identifies significantly more distant interactions than the other techniques (Fig. 2b, Additional file 3: Figure S1a, Wilcoxon rank-sum test *p* value = 1.1E−223). Additionally, we note that the eQTL and crisprQTL interactions all have maximum distances of 1 Mb (Additional file 3: Figure S1a) because the original studies only tested SNPs within 1 Mb of each gene.

**Table 1:** Genomic interaction dataset

Assay		Reference
3D chromatin interactions		
Hi-C	High-resolution in situ Hi-C: identifies chromatin loops anchored by convergent CTCF binding sites	[14]
RNAPII ChIA-PET	Chromatin interaction analysis by paired-end tag sequencing targeting RNAPII: identifies chromatin interactions enriched for RNAPII binding	[16]
CTCF ChIA-PET	Chromatin interaction analysis by paired-end tag sequencing targeting CTCF: identifies chromatin interactions enriched for CTCF binding	[16]
CHi-C	Promoter capture Hi-C: identifies chromatin interactions between promoters and other loci	[17]
Genetic interactions		
eQTLs	Expression quantitative trait loci: identifies genetic variants correlated with changes of gene expression of individuals in a human population	[18, 19]
CRISPR/Cas9 perturbations		
crisprQTLs	Identifies loci that when targeted with CRISPR/Cas9 correlate with changes in gene expression measured in single cells	[20]

We then compared the gene expression of the positive pairs among the six GM12878/LCL datasets (Fig. 2c). Overall, the genes in the GEUVADIS eQTL pairs exhibited the highest median expression (median = 10.9 transcripts per million sequenced reads, or TPM; Wilcoxon rank-sum test *p* = 1E−3), while the genes in the CHi-C pairs presented the lowest median expression levels (median = 0.24 TPM, *p* = 7E− 39). When we removed ambiguous pairs, gene expression increased significantly for all four chromatin interaction datasets (Additional file 3: Figure S1b), suggesting that some of the ambiguous pairs were false positives. We observed similar increases in gene expression upon the removal of ambiguous pairs in other cell types for which we had RNA-seq data (Additional file 3: Figure S1c-e). Without the ambiguous pairs, the RNAPII ChIA-PET pairs showed comparable expression to the GEUVADIS eQTL pairs. The enrichment for RNAPII in the ChIA-PET protocol may preferentially identify interactions that involve higher RNAPII activity and higher gene expression. The K562 crisprQTL pairs presented the highest overall median expression of 26.4 TPM. We expected to observe high expression for the eQTL and crisprQTL datasets because these interactions can only be detected for genes that are expressed in the respective biosamples.

We also observed significant differences in the CTCF ChIP-seq signals at cCREs-ELS between the BENGI datasets: cCREs-ELS in CTCF ChIA-PET pairs and Hi-C pairs showed significantly higher CTCF signals than cCREs-ELS in the other datasets (Wilcoxon rank-sum test *p* < 3.7E− 9, Fig. 2d, Additional file 2: Table S2b). Similarly, these pairs were enriched for components of the cohesin complex such as RAD21 and SMC3 (Additional file 2: Table S2b). This enrichment for CTCF was biologically consistent, as CTCF was the target in the ChIA-PET experiment, and Hi-C loops are enriched for convergent CTCF binding sites [14].

Finally, we tallied the number of linked genes for each cCRE-ELS. Across all BENGI datasets, the majority of cCREs-ELS were linked to just one target gene (Fig. 2e, Additional file 2: Table S2c). As expected, this trend was more pronounced for 3D chromatin datasets without ambiguous pairs (on average, 84% of cCREs-ELS were paired with only one gene, *p* < 3.3E−5). With or without ambiguous pairs, a lower percentage of cCREs-ELS in CHi-C pairs was paired with just one gene (19% of all pairs and 55% of unambiguous pairs) than in the other BENGI datasets (*p* < 3.1E− 75). This observation, along with the lower average expression of the linked genes (Fig. 2c), suggests that some of the CHi-C pairs were either false positives or captured interactions between cCREs-ELS and genes that are yet to be expressed.

These analyses suggested that the various experimental techniques whose results formed the basis of the BENGI datasets capture different classes of genomic interactions. Because we do not have a complete understanding of which experimental techniques are best able to capture bona fide enhancer-gene interactions, we propose that computational methods (Table 2) should be evaluated on the entire collection of these BENGI datasets to provide a comprehensive understanding of their performance.

**Table 2:** Computational methods for target gene prediction

Method	Description	Reference
Unsupervised methods		
Distance	Ranks pairs by inverse linear distance	
DNase-DNase	Calculates the Pearson correlation coefficient between the DNase signals at enhancers and promoters across 32 cell-type categories.	[22]
DNase-expression	Calculates the Pearson correlation coefficient between the normalized DNase signals at enhancers and normalized gene expression levels measured by microarray across 112 cell types.	[23]
GeneHancer	Cell-type agnostic predictions based on co-expression correlations, CHi-C interactions, eQTLs, and genomic distance	[31]
Average-rank	Combines the distance and DNase-expression methods by averaging the rank of for each prediction between the two methods	
Supervised methods		
PEP-motif	*Features:* frequency of motif instances at enhancers and promoters	[28]
	*Classifier:* Gradient boosting (XGB package)	
TargetFinder	*Features:* Cell-type-specific epigenomic signals (ChIP-seq, DNase, CAGE, etc.) at enhancers, promoters, and the intervening window between enhancers and promoters.	[27]
	*Classifier:* Gradient boosting (scikit learn)	

### A baseline method of target gene prediction using genomic distance

Using the BENGI datasets, we evaluated a simple *closest gene* method for target gene prediction: a cCRE-ELS was assigned to its closest gene in terms of linear distance, computed by subtracting the genomic coordinates of the cCRE and the nearest TSS. All BENGI datasets, despite interaction type, had highly similar ELS-gene distance distributions (Additional file 3: Figure S1f). We tested this method using two gene sets, consisting of all genes or all protein-coding genes annotated by GENCODE V19, by evaluating precision and recall on the basis of each BENGI dataset. The use of protein-coding genes invariably resulted in better performance than the use of all genes (50% better on average over all 21 datasets across cell types; Additional file 2: Table S2d); thus, we used protein-coding genes for all subsequent analyses with this method.

The *closest gene* method worked best for crisprQTL pairs (precision = 0.67 and recall = 0.60), followed by ChIA-PET RNAPII pairs (precision = 0.66 and recall = 0.31 averaged across cell lines). The method performed worst for Hi-C pairs, with an average precision of 0.19 and an average recall of 0.12. These results are consistent with our statistical analyses described above, which revealed that crisprQTL and RNAPII ChIA-PET pairs were enriched in gene-proximal interactions, while Hi-C pairs tended to identify more distal interactions.

**Fig. 3:**
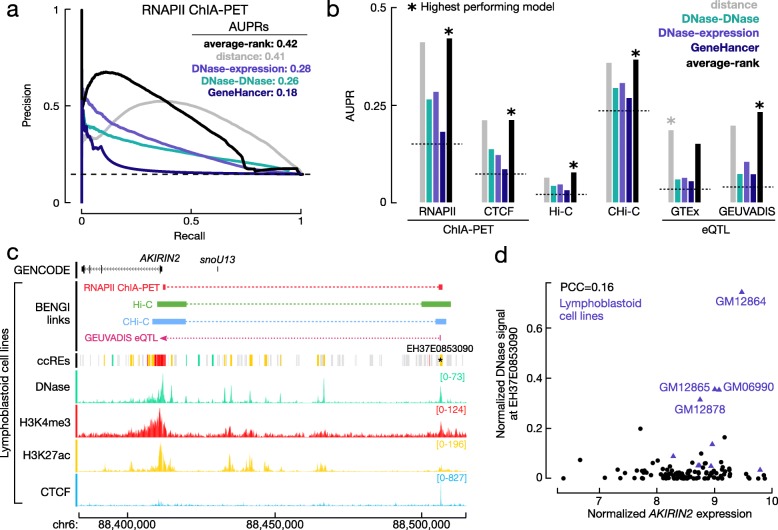
Evaluation of unsupervised methods for predicting cCRE-gene pairs. **a** Precision-recall (PR) curves for four unsupervised methods evaluated on RNAPII ChIA-PET pairs in GM12878: distance between cCREs-ELS and genes (gray), DNase-DNase correlation by Thurman et al. (green), DNase-expression correlation by Sheffield et al. (purple), and the average rank of the distance and the DNase-expression method (black). The areas under the PR curve (AUPRs) for the four methods are listed in the legend. The AUPR for a random method is indicated with a dashed line at 0.15. **b** The AUPRs for the four unsupervised methods are computed for each of the six benchmark datasets from LCLs. **c** Genome browser view (chr6:88,382,922-88,515,031) of epigenomic signals and positive BENGI links (RNAPII ChIA-PET in red, Hi-C in green, CHi-C in blue, and GEUVADIS eQTL in pink) connecting the EH37E0853090 cCRE (star) to the *AKIRIN2* gene. **d** Scatter plot of normalized *AKIRIN2* expression vs. the normalized DNase signal at EH37E0853090 as calculated by Sheffield et al. (Pearson correlation coefficient = 0.16). Although *AKIRIN2* is highly expressed across many tissues, EH37E0853090 presents high DNase signals primarily in lymphoblastoid cell lines (purple triangles), resulting in a low correlation

For comparison with other enhancer-gene prediction methods, we adapted the *closest gene* method to a quantitative ranking scheme where we ordered cCRE-gene pairs by the distance between the cCRE-ELS and the gene’s closest TSS. For each BENGI dataset, we evaluated the overall performance of the resulting *distance* method by calculating the area under the precision-recall curve (AUPR). Accordingly, the *distance* method exhibited the highest AUPR (0.41) for RNAPII ChIA-PET pairs and the lowest AUPR (0.06) for Hi-C pairs (Fig. 3a,b, Additional file 3: Figure S2b, Additional file 4: Table S3). Since the distance method is cell-type independent and does not require any experimental data, we considered it as the baseline method for comparing all enhancer-gene prediction methods.

### Correlation-based approaches perform worse than the distance method

We next evaluated the performance of two correlation-based methods with the BENGI datasets: a method based on correlating the DNase signals at predicted enhancers with the DNase signals at TSSs across a panel of biosamples [22] and a method based on correlating DNase signals with gene expression [23]. Both the DNase-DNase and DNase-expression methods outperformed random predictions for all 21 BENGI datasets, with average AUPR values of 0.10 and 0.12 vs. 0.07, respectively, but the differences were modest (Additional file 3: Figure S2; Additional file 4: Table S3). As previously demonstrated [22], positive pairs presented significantly higher correlations under both methods than negative pairs in all datasets (Additional file 3: Figure S2); however, the relative rankings of these correlations were mixed and did not completely segregate positive from negative pairs. The DNase-expression method significantly outperformed the DNase-DNase method for all but two BENGI datasets (Wilcoxon signed-rank test *p* = 6.7E−5), with an average AUPR increase of 29% (Additional file 2: Table S2).

We then evaluated the performance of the GeneHancer prediction model, via an integration of four types of enhancer annotations, including an earlier version of our cCREs, to generate a collection of candidate enhancers [31]. These candidate enhancers were then linked to genes by integrating co-expression correlations, eQTLs, CHi-C data, and genomic distance. Because the authors used eQTLs and CHi-C from the same data sources as those in BENGI to build the GeneHancer model, we only evaluated the performance of the model on the ChIA-PET, Hi-C, and crisprQTL pairs. While the GeneHancer predictions were better than random predictions, the differences were extremely modest (average improvement of 0.01 in AUPR). The GeneHancer predictions also had a much lower overall recall than the correlations methods (on average 8% compared to 100% and 76% for DNase-DNase and DNase-expression respectively). Even for these limited sets of predictions, GeneHancer never outperformed the DNase-expression model and only outperformed the DNase-DNase model for crisprQTLs (Additional file 3: Figure S3).

Ultimately, the distance method substantially outperformed the two correlation-based methods and the GeneHancer predictions: distance was better than DNase-DNase for all 21 datasets (average AUPR increase of 127%; *p* = 1.9E−6; Additional file 2: Table S2), better than DNase-expression for 17 datasets (average AUPR increase of 77%; *p* = 1.6E−4), and better than GeneHancer predictions for all datasets (average AUPR increase of 256%; *p* = 9.5E−7). The PR curves of the distance method and the two correlation-based methods for the RNAPII ChIA-PET pairs are shown in Fig. 3a. For the first 25 k predictions, the distance method presented a similar precision to the DNase-DNase method and lower precision than the DNase-expression method, but when more predictions were made, the distance method substantially outperformed both correlation-based methods and achieved a much higher AUPR (0.41 vs. 0.28 and 0.26). We observed this crossover of PR curves in other non-QTL datasets as well (Additional file 3: Figure S2); thus, we integrated the distance and DNase-expression methods by averaging their ranks for the same prediction. Notably, this average-rank method showed high precision for its top-ranked predictions (Fig. 3a) and achieved higher AUPRs than the other methods for all 13 datasets except for GTEx eQTL pairs, with an average AUPR increase of 17% over the distance method for these datasets (Fig. 3b, Additional file 2: Table S2). For the eight GTEx eQTL datasets, the distance method remained the best approach, showing an 18% higher AUPR on average than the second-best method, average rank (Additional file 2: Table S2).

We asked why correlation-based methods performed poorly for predicting enhancer-gene pairs. One particular example is highlighted in Fig. 3 c, d. cCRE-ELS EH37E0853090 was paired with the *AKIRIN2* gene by RNAPII ChIA-PET, Hi-C, CHi-C, and a GEUVADIS eQTL (Fig. 3c). However, this pair was poorly ranked by both correlation-based methods (correlation coefficients: *r* = 0.03 and 0.16 for DNase-DNase and DNase-expression, respectively). *AKIRIN2* was highly expressed in most surveyed cell types (median normalized expression of 8.5 vs. background of 4.7 RPKM, Additional file 3: Figure S4a), and its promoter exhibited a high DNase signal (signal ≥ 50) for each of the DNase-seq groups (Additional file 3: Figure S4b). However, EH37E0853090 only presented high DNase signals in four cell types, which were all lymphoblastoid cell lines, suggesting that this enhancer was primarily active in the B cell lineage. The ubiquitous expression of *AKIRIN2* and the cell-type-specific activity of EH37E0853091 resulted in a low correlation (Fig. 3d, Additional file 3: Figure S4b). In general, TSS-overlapping cCREs (cCREs-TSS) are active in many more biosamples than distal cCREs-ELS (median of 92 vs. 46 biosamples, *p* = 3.6E− 264, Additional file 3: Figure S4c-d). In summary, because the epigenomic signals at cCREs-ELS are far more cell type specific than the epigenomic signals at TSSs and gene expression profiles, correlation across biosamples is a poor method for detecting enhancer-gene pairs.

### Supervised methods outperform baseline methods upon cross-validation

We tested two supervised machine-learning methods that were reported to perform well in the original publications on the methods: TargetFinder, which uses epigenomic signals such as histone mark ChIP-seq, TF ChIP-seq, DNase-seq in the corresponding cell types as input features, and PEP-motif, which uses the occurrence of TF sequence motifs as features. Xi et al. subsequently revealed that the original implementation of cross-validation (CV) by TargetFinder and PEP-motif allowed the assignment of enhancer-gene pairs from the same genomic loci to different CV groups, which led to sharing of training and testing data, overfitting of their models, and inflated performance [29]. Thus, we implemented the chromCV method to ensure that pairs from the same chromosome were always assigned to the same CV group (Fig. 1e; “Methods”).

**Fig. 4:**
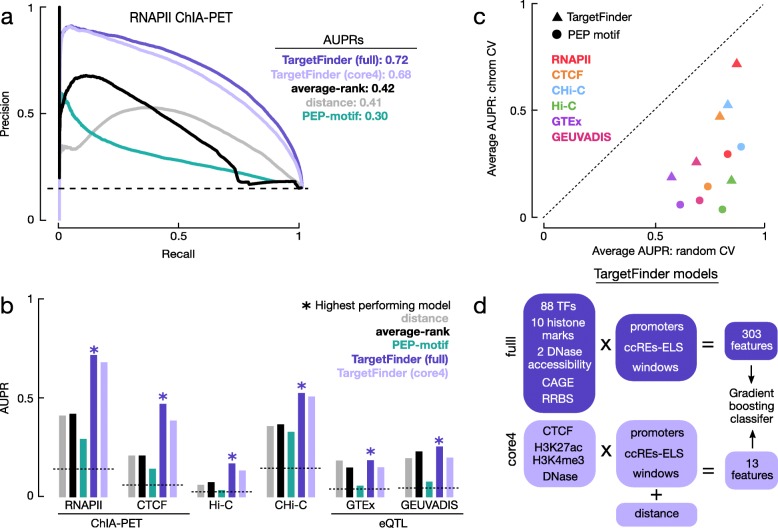
Evaluation of supervised learning methods for predicting cCRE-gene pairs. **a** PR curves for three supervised methods evaluated using RNAPII ChIA-PET pairs in GM12878: PEP-motif (green) and two versions of TargetFinder (full model in darker blue and core model in lighter blue). For comparison, two unsupervised methods presented in Fig. 3 (the distance (gray) and average-rank (black) methods) are also shown along with the AUPR for a random method (dashed line at 0.15). The AUPRs for the methods are listed in the legend. **b** AUPRs for the three supervised methods, two unsupervised methods, and a random approach, colored as in **a**, for each of the six BENGI datasets from LCLs. **c** Scatter plot of AUPRs for TargetFinder (triangles) and PEP-motif (circles) across the BENGI datasets evaluated using 12-fold random CV (*X*-axis) vs. chromosome-based CV (*Y*-axis). The diagonal dashed line indicates *X* = *Y*. **d** Schematic diagram for the full and core4 TargetFinder models

We first tested these two supervised methods on the six BENGI datasets in GM12878 because there were a large number of epigenomic datasets for this cell type that could be used as features to train the methods. Although PEP-motif performed better than random, it underperformed the distance method for all GM12878 pairs and was far worse than the average-rank method pairs (Fig. 4a, b; Additional file 2: Table S2b). In contrast, TargetFinder outperformed the average-rank method for all six datasets, with an average AUPR improvement of 66% (Fig. 4a, b; Additional file 2: Table S2), but the AUPRs were still low, especially for the Hi-C (0.17) and eQTL datasets (0.19 and 0.26).

Because the results of TargetFinder and PEP-motif upon our chromCV implementation were worse than the original published results for these methods, we also implemented a randomized 12-fold CV method as described in the original publications to test whether we could reproduce their results. Indeed, we observed large performance decreases for the chromCV method with respect to the original CV method (Fig. 4c), suggesting that overfitting was a source of inflated performance. PEP-motif presented a more substantial decrease in performance (average AUPR decrease of 80%) than TargetFinder (average AUPR decrease of 51%), likely because PEP-motif added 4 kb of padding on both sides of each enhancer, increasing the chance of overlapping training and testing data. Although PEP-motif and TargetFinder used Hi-C loops as the gold standard in their original analyses, both methods showed the largest performance decreases for the BENGI GM12878 Hi-C pairs (AUPR decrease of 95% for PEP-motif and 80% for TargetFinder). This analysis further highlights the utility of a carefully designed benchmark to prevent overfitting of supervised models.

Our implementation of TargetFinder in GM12878 cells involved 101 epigenomic datasets, including ChIP-seq data for 88 TFs, resulting in a total of 303 input features (Fig. 4d). However, such extensive TF ChIP-seq data were not available for other biosamples; thus, we also trained TargetFinder models using only distance and four epigenomic features: DNase, H3K4me3, H3K27ac, and CTCF data, which we refer to as the core4 TargetFinder models. While the core4 models exhibited an average AUPR reduction of 23% compared with the respective full models across the 13 BENGI datasets (Fig. 4a, b; Additional file 4: Table S3), they still outperformed the distance and average-rank methods for all datasets. Of particular note were the IMR-90 Hi-C pairs, which presented the greatest decrease in performance between the full and core4 TargetFinder models, with an AUPR reduction of 0.29 (81%). We observed similar large decreases in performance across all four variations of the IMR-90 Hi-C pairs. We also trained core3 models for the biosamples without CTCF data, and they showed an average AUPR reduction of 34% compared with the respective full models across the 13 BENGI datasets. For the seven GTEx eQTL datasets from tissues, these core3 models did not outperform the distance or average-rank models.

Overall, TargetFinder’s performance on the RNAPII and CTCF ChIA-PET pairs was markedly higher than its performance on other BENGI datasets. These datasets were the only two benchmarks of 3D chromatin interactions mediated by specific TFs. When we analyzed the feature-importance scores (i.e., Gini importance) from TargetFinder’s GBM model, we found that RNAPII and CTCF ChIP-seq signals at promoters had the highest importance in the respective models. To further dissect the features contributed to TargetFinder’s performance, we ran the algorithm on a subset of positive and negative pairs (1:2 ratio of positives to negatives) and three selections of positive and negative pairs that were matched for (i) only promoter inclusion, (ii) only distance, and (iii) promoter inclusion and distance (for promoter distance, see “Methods”). For all four subsets, the full TargetFinder still outperformed all other methods (Additional file 5: Table S4e); however, compared to the 1:2 ratio set (average AUPR = 0.86), performance was lower for the distance-matched and promoter-matched sets (average AUPR = 0.74 and 0.69) and was the lowest for the promoter-distance-matched sets (average AUPR = 0.61). We observed similar patterns with the TargetFinder core4 and core3 though the relative drop in performances was much larger—average decreases in AUPR of 0.25 for full model, 0.28 for core4 model, and 0.32 for core-3 model. Particularly, for the core3 CTCF ChIA-PET promoter-distance model, which does not include CTCF as a feature, we observed an AUPR of 0.43, a 0.30 reduction in AUPR compared to the 1:2 ratio pairs, and only a 0.03 improvement in AUPR over the DNase-DNase correlation method. These results suggest that differences in RNAPII/CTCF ChIP-seq signal and distance between positive and negative pairs contribute to TargetFinder’s ability to successfully predict cCRE-ELS-gene pairs.

### TargetFinder exhibits moderate performance across different cell types

The most desirable application of a supervised method is to train the model in a biosample with 3D chromatin or genetic interaction data and then use the model to make predictions in another biosample without such data. Thus, we tested the TargetFinder core4 and core3 models for such application to the ChIA-PET, Hi-C, CHi-C, and GTEx eQTL datasets, readjusting our chromCV method to prevent overfitting [32] (see “Methods”).

**Fig. 5:**
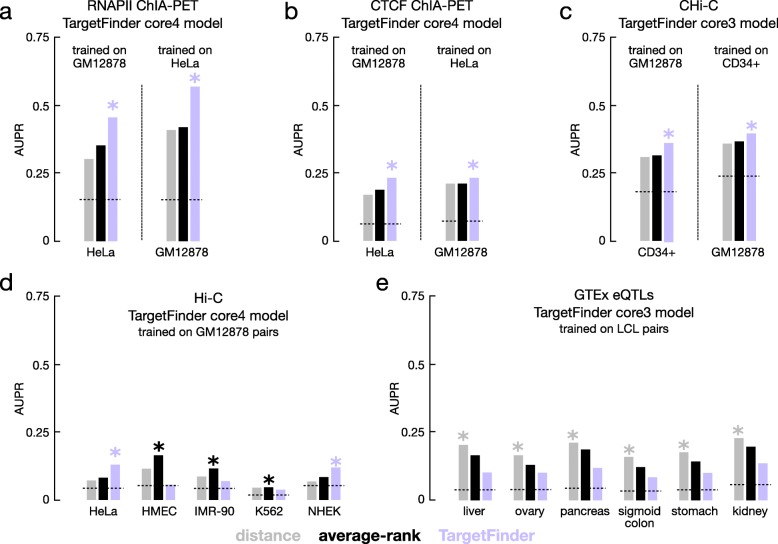
Evaluation of supervised learning methods trained in one cell type and tested in another cell type. AUPRs for the distance (gray), average-rank (black), and TargetFinder core4 (purple) methods across **a** RNAPII ChIA-PET, **b** CTCF ChIA-PET, **c** CHi-C, **d** Hi-C, and **e** GTEx eQTL pairs. The cell type used for training is indicated in the panel title, and the cell type used for testing is indicated on the *X*-axis. The best-performing method for each dataset is indicated by a star, and random performance is indicated with a dashed line

As expected, the cross-cell-type models performed worse than the same-cell-type models, but their performance varied compared with the unsupervised distance and average-rank methods. For the CHi-C and RNAPII ChIA-PET datasets, all tested cross-cell-type TargetFinder models outperformed the distance and average-rank methods for both tested cell types (GM12878 vs. HeLa and GM12878 vs. CD34+), with average AUPR increases of 32% and 12%, respectively (Fig. 5a,b, Additional file 6: Table S5). For CTCF ChIA-PET, the core3 model trained on HeLa cells did not outperform the unsupervised methods for predicting GM12878 pairs (AUPR = 0.15 vs 0.21), but the models trained on GM12878 and the core4 model trained on HeLa did slightly outperform the unsupervised methods for predicting HeLa pairs and GM12878 pairs respectively (average AUPR increase of 7% Fig. 5c, Additional file 6: Table S5). The results for the Hi-C datasets were mixed. Among the 60 cross-cell-type models tested, 12 outperformed the distance and average-rank methods. Specifically, the model trained on GM12878 only outperformed the distance and average-rank methods for predicting HeLa or NHEK pairs (Fig. 5d, Additional file 6: Table S5), with an average 50% increase in performance. The model trained on IMR-90 never outperformed the distance and average-rank methods, and for the prediction of HMEC, IMR-90, and K562 pairs, none of the cross-cell-type models outperformed the distance or average-rank methods (Additional file 6: Table S5). These results were consistent across the fixed ratio pairs as well. Finally, none of the cross-cell-type models outperformed the distance method for the GTEx datasets; the distance method was the highest-performing model for all GTEx datasets (Additional file 6: Table S5).

## Discussion

Here, we have presented BENGI, a benchmark comprising cCRE-ELS-gene pairs, curated through the integration of the Registry of cCREs and genomic interaction datasets. We used BENGI to evaluate four published computational methods for target gene prediction that represent most of the widely used approaches in the field while surveying orthogonal dimensions: correlation methods survey across the biosample dimension, while supervised machine-learning methods such as TargetFinder survey across the assay dimension. We found that the two correlation-based, unsupervised methods significantly underperformed the baseline distance method, while one of the two supervised methods examined, TargetFinder, significantly outperformed the distance method when trained and tested within the same cell type by cross-validation. Although TargetFinder outperformed the distance method for all BENGI datasets, the AUPRs of the TargetFinder models were generally still low (0.07–0.72). In particular, TargetFinder performed best on ChIA-PET pairs; however, the performance substantially decreased when the positive and negative pairs were matched for their distributions of RNAPII/CTCF ChIP-seq signals at promoters and cCRE-ELS-gene distances. Thus, these features are the main contributors to TargetFinder’s higher performance on ChIA-PET datasets than other BENGI datasets. The other supervised method, PEP-motif, significantly underperformed the distance method, suggesting that the frequencies of TF motifs at enhancers and promoters are not sufficiently predictive of genomic interactions. When trained and tested in different cell types, TargetFinder performed better than the distance method for some BENGI datasets, albeit by a much smaller amount. Overall, there is much room for improvement for all of these methods, indicating that target gene prediction remains a challenging problem. BENGI datasets can be used by the community to tackle this problem while avoiding overfitting issues such as those identified for TargetFinder and PEP post publication [29, 30].

Our analyses highlight the differences between the genomic interactions identified by various experimental techniques (Table 1). For the same biosample (e.g., LCLs), the BENGI datasets generated by the same technique shared ~ 40% of their pairs (e.g., between RNAPII and CTCF ChIA-PET and between GEUVADIS and GTEx eQTLs), but the overlap between the datasets generated by different techniques were typically lower than 25% and could be as low as 1% (e.g., between eQTL and Hi-C). The BENGI datasets also differed significantly in terms of enhancer-gene distance and the enrichment of epigenomic signals at enhancers and TSSs. Thus, we still do not have a comprehensive understanding of the factors that regulate enhancer-gene interactions, and these different experimental techniques may capture different subsets of interactions.

Overall, all computational methods evaluated presented difficulty in predicting Hi-C pairs; even for the fixed ratio datasets, the Hi-C pairs consistently exhibited the lowest overall performance. This could be due to the technical challenges of calling Hi-C loops or the biological roles of these loops. For example, it has been noted that the detection of Hi-C loops requires care, and different loop-calling methods can produce markedly different results [33]. Additionally, recent results from the Aiden lab demonstrated that gene expression did not change upon loop disruption via knocking out the key protein CTCF using a degron system [34]. This finding may suggest that these CTCF Hi-C loops may play specific biological roles and may only represent a small subset of enhancer-gene interactions that have different properties compared to the other interactions.

Although the correlation-based methods did not outperform the distance method, the DNase-expression method did augment the distance method when combined with it. Furthermore, because correlation-based methods and supervised machine-learning methods survey orthogonal dimensions (biosample vs. assay), one promising future direction will be to combine these two types of approaches. For such future work to be fruitful, it will be beneficial to understand the differences in performance between the two correlation-based methods because the DNase-expression correlation method consistently outperformed the DNase-DNase correlation method. Several factors could contribute to this increased performance. First, gene expression may be a better readout for enhancer-gene interactions than a promoter’s chromatin accessibility, although these two features are correlated (average Pearson correlation *r* = 0.68). Second, for the DNase-expression method, Sheffield et al. generated normalized, batch-corrected matrices for the DNase-seq and gene expression data, while the DNase-DNase method used a read depth-normalized signal without any additional processing. To avoid imprecision in reimplementation, we downloaded these exact input datasets from the original publications (i.e., the exact normalized matrices for the DNase-expression method and the ENCODE2-processed DNase-seq bigWigs for the DNase-DNase method). The Sheffield et al. normalization technique may correct for outliers and batch effects, which would otherwise lead to spurious correlations impacting performance. Third, the DNase-DNase method merged 79 cell types into 32 groups based on cell type similarity. While this grouping may correct an uneven survey of the biosample space, it may lead to lower overall correlations for cell-type-specific interactions. We highlighted one such case involving the LCL-specific EH37E0853090-AKIRIN2 interaction, where the DNase-DNase method reported a correlation of 0.03, and the DNase-expression method reported a correlation of 0.12. The low correlation calculated by the DNase-DNase method was due to the combination of the four LCLs in one group, reducing the statistical power (Additional file 3: Figure S4b). These possible explanations should be carefully considered when designing future correlation-based and combined methods. Additionally, although these correlation-based methods did not perform well on the BENGI datasets, they may present better predictive power when used on curated sets of biosamples such as those obtained across embryonic development or cell differentiation. As we expand the number of cell types and tissues covered by BENGI, we hope to test these methods to evaluate their performance systematically.

Finally, we developed BENGI using an enhancer-centric model, as we were motivated by the Registry of cCREs. We hope to expand upon this approach to include a gene-centric model (i.e., for a given gene, determine the interacting enhancers) for future developments. Additionally, though BENGI datasets currently span 13 biosamples, the majority of the gene-ELS pairs derived from GM12878 or LCLs because these cells have been extensively profiled. Therefore, users of the benchmark should be cognizant that not all biosamples are profiled equally. Furthermore, the remaining BENGI datasets all derived from cell lines or heterogeneous tissues, none from primary cells. We will increase the representation of primary cells in our benchmark as soon as 3D chromatin and genetic interaction data on primary cells become available. We also plan to expand BENGI to include more functionally tested datasets such as the crisprQTLs as these results are published.

## Conclusions

Precise and accurate identification of enhancer-gene links in a cell-type-specific manner remains a major challenge. Systematic comparisons using the BENGI datasets enabled us to identify the pitfalls in the current repertoire of computational methods, such as correlation-based approaches and the more complex, tree-based supervised algorithms. BENGI will aid the development of future enhancer-gene prediction models and improve our understanding of how regulatory elements control gene expression and ultimately the role that regulatory elements play in human diseases.

## Methods

### Data acquisition

#### ChIA-PET

We downloaded the following ChIA-PET clusters generated by the Ruan lab [16] from the NCBI Gene Expression Omnibus (GEO) under accession number GSE72816.

GSM1872886_GM12878_CTCF_PET_clusters.txt

GSM1872887_GM12878_RNAPII_PET_clusters.txt

GSM1872888_HeLa_CTCF_PET_clusters.txt

GSM1872889_HeLa_RNAPII_PET_clusters.txt

We filtered each set of clusters by selecting ChIA-PET links that were supported by at least four reads (column 7 ≥ 4).

#### Hi-C loops

We downloaded the following Hi-C loops generated by the Aiden lab [14] from GEO under accession number GSE63525.

GSE63525_GM12878_primary+replicate_HiCCUPS_looplist.txt

GSE63525_HMEC_HiCCUPS_looplist.txt.gz

GSE63525_HeLa_HiCCUPS_looplist.txt.gz

GSE63525_IMR90_HiCCUPS_looplist.txt.gz

GSE63525_K562_HiCCUPS_looplist.txt.gz

GSE63525_NHEK_HiCCUPS_looplist.txt.gz

We did not perform any additional filtering on these loops.

#### CHi-C

We downloaded the following CHi-C interactions generated by the Osborne lab [17] from ArrayExpress under accession number E-MTAB-2323.

TS5_GM12878_promoter-other_significant_interactions.txt

TS5_CD34_promoter-other_significant_interactions.txt

We filtered each set of interactions selecting CHi-C links by requiring a log(observed/expected) value greater than ten (column 11 > 10).

#### eQTLs

We downloaded cis-eQTLs from the GEUVADIS project:

ftp://ftp.ebi.ac.uk/pub/databases/microarray/data/experiment/GEUV/E-GEUV-1/analysis_results/

EUR373.gene.cis.FDR5.all.rs137.txt

We downloaded single-tissue cis-eQTLs (GTEx_Analysis_v7_eQTL.tar.gz) from the GTEx Portal https://gtexportal.org/home/datasets. We used the following files:

Cells_EBV-transformed_lymphocytes.v7.signif_variant_gene_pairs.txt

Colon_Sigmoid.v7.signif_variant_gene_pairs.txt

Liver.v7.signif_variant_gene_pairs.txt

Ovary.v7.signif_variant_gene_pairs.txt

Pancreas.v7.signif_variant_gene_pairs.txt

Stomach.v7.signif_variant_gene_pairs.txt

Thyroid.v7.signif_variant_gene_pairs.txt

#### CRISPR perturbations

We downloaded crisprQTL data from Gasperini et al. [20] and mapped the reported genes to those annotated in GENCODE V19 and intersected the reported enhancer coordinates with cCREs-ELS in K562. A total of 4937 of the tested enhancers (85%) overlapped a K562 cCRE-ELS.

### Defining cCREs-ELS

We used cCREs-ELS from V1 of the ENCODE Registry of cCREs available on the ENCODE portal found under the accessions provided in Additional file 1: Table S1a. We selected all cCREs-ELS (RGB color code 255,205,0) that were distal (i.e., greater than 2 kb from an annotated TSS, GENCODE v19).

### Defining cCRE-gene pairs

We created cCRE-gene pairs using the *Generate-Benchmark.sh*. script, which is available on GitHub [35].

#### 3D chromatin interactions (ChIA-PET, Hi-C, and CHi-C)

Using bedtools intersect (v2.27.1), we intersected the anchors of the filtered links (see above) with cCREs-ELS that were active in the same biosample. We retained all links with an anchor that overlapped at least one cCREs-ELS and with the other anchor within ± 2 kb of a GENCODE V19 TSS. We tagged all links with an anchor within ± 2 kb of the TSSs of multiple genes as ambiguous pairs and created a separate version of each dataset with these links removed.

#### Genetic interactions (eQTLs)

For eQTLs, we retrieved the location of each reported SNP from the eQTL file and intersected these loci with cCREs-ELS that were active in the same tissue type using bedtools intersect. We then paired the cCRE-ELS with the gene linked to the SNP. We only considered SNPs that were directly reported in each of the studies; we did not expand our set using linkage disequilibrium due to the mixed populations surveyed by GTEx.

#### CRISPR/dCas-9 (crisprQTLs)

For crisprQTLs, we intersected the reported positive enhancers with cCREs in K562 using bedtools intersect. We then paired the cCRE-ELS with the gene linked to the reported enhancer.

#### Generation of negative pairs

To generate negative pairs, we calculated the 95th percentile of the distances of positive cCRE-gene pairs for each dataset, with distance defined as the linear distance between the cCRE-ELS and the closest TSS of the gene using bedtools closest. For each cCRE-ELS among the positive cCRE-gene pairs that fell within this 95th percentile, we considered all other genes within the 95th percentile distance cutoff as negatives. Because our model is enhancer-centric, the same promoter may belong to both positive and negative sets, paired with different enhancers. For datasets with ambiguous links removed (ChIA-PET, Hi-C, and CHi-C), we also excluded genes in these ambiguous pairs as negatives. For the fixed ratio datasets, we also excluded genes that were in the positive pairs for the cCREs-ELS in other BENGI datasets before randomly selecting the negatives. If a cCRE-ELS exhibited fewer than four negative pairs, then it was excluded from this fixed ratio set.

#### Assignment of chromosome CV

For each BENGI dataset, we calculated the number of cCRE-gene pairs on each chromosome and assigned chromCV groups accordingly. The chromosome with the most pairs (often chr1) was assigned its own group. Then, we iteratively took the chromosome with the most and fewest pairs and combined them to create one CV group. In total, the 23 chromosomes (1–22, X) were assigned to 12 CV groups.

### Characterization of BENGI datasets

#### Clustering of dataset overlap

For each pairwise combination of the GM12878/LCL BENGI datasets, we calculated the overlap coefficient of positive cCRE-gene pairs. Then, using *hclust*, we performed hierarchical clustering with default parameters.

#### Gene expression

For biosamples with matching RNA-seq data, we downloaded corresponding RNA-seq data from the ENCODE portal (accessions provided in Additional file 1: Table S1b, Additional file 3: Figure S1). For each gene, we calculated the average TPM between the two experimental replicates. To test whether there was a significant difference between BENGI datasets with or without ambiguous pairs, we used a Wilcoxon test.

#### ChIP-seq signals

For cCREs-ELS in each positive pair across the GM12878 and LCL BENGI datasets, we calculated the average ChIP-seq signal for 140 transcription factors and DNA-binding proteins. We downloaded the ChIP-seq signal from the ENCODE portal (accession available in Additional file 2: Table S2b) and used UCSC’s *bigWigAverageOverBed* to calculate the average signal across each cCRE. For each BENGI dataset, we then reported the average signal for all cCREs.

### Implementation of cCRE-gene prediction methods

#### Closest-gene method

We identified the closest TSS to each cCRE-ELS using bedtools closest and GENCODE V19 TSS annotations. We compared two options: use of the full set of GENCODE TSSs (with problematic annotations removed) or use of only protein-coding GENCODE TSSs. To evaluate performance, we calculated the overall precision and recall for each BENGI dataset (Script: Closest-Gene-Method.sh).

#### Distance method

For each cCRE-gene pair, we calculated the linear distance between the cCRE-ELS and the gene’s nearest TSS. To rank these pairs, we took the inverse (1/distance) and calculated the area under the precision-recall curve (AUPR) using a custom R script that uses the PROCR library (Script: Run-Distance-Method.sh).

#### *DNase-DNase* correlation method

We used the same DNase-seq datasets as Thurman et al. employed for their DNase-DNase method. We downloaded these legacy datasets generated during ENCODE Phase 2 from the UCSC genome browser. For each cCRE-gene pair, we curated a set of cCREs-TSS by determining the closest cCRE for each TSS of the gene. We then calculated the average DNase signal across the nucleotide positions in the cCRE-ELS and cCRE-TSS for each DNase dataset. For similar cell types, as determined by Thurman et al., we averaged the DNase signal among these similar cell types in each of the 32 groups to generate 32 values for each cCRE-ELS and cCRE-TSS. We then calculated the Pearson correlation coefficient (PCC) for each cCRE-ELS and cCRE-TSS pair. If a gene was annotated with multiple TSSs, we selected the highest PCC among all the cCRE-ELS and cCRE-TSS comparisons. We ranked the predictions by their PCC and calculated the AUPR using the PROCR library (Script: Run-Thurman.sh).

#### *DNase-expression* correlation method

To match the legacy data and normalization methods originally used by previous investigators [23], we downloaded normalized counts across 112 cell types for DNase-hypersensitive sites or DHSs (*dhs112_v3.bed*) and genes (*exp112.bed*) from http://big.databio.org/papers/RED/supplement/. We intersected each cCRE-ELS with the DHSs previously curated [23]. If a cCRE overlapped with more than one DHS, we selected the DHS with the strongest signal for the cell type in question (i.e., the DHS with the strongest signal in GM12878 for GM12878 cCREs-ELS). For each cCRE-gene pair, we then calculated the Pearson correlation coefficient using the 112 normalized values provided in each matrix. cCRE-gene pairs that did not overlap with a DHS or did not have a matching gene in the expression matrix were assigned a score of − 100. (Script: Run-Sheffield.sh).

#### PEP-motif

We reimplemented PEP-motif to run on our cCRE-gene pairs with chromCV. Similar to Yang et al., we calculated motif frequency using FIMO [36] and the HOCOMOCO database (v11 core, [37]). We also added ± 4 kb of padding to each cCRE-ELS as originally described. We concatenated cross-validation predictions and calculated AUPR values using PROCR (Script: Run-PEPMotif.sh).

#### TargetFinder

We reimplemented TargetFinder to run on our cCRE-gene pairs with chromCV. For features, we used the identical datasets described by Whalen et al. for each cell type. We concatenated the cross-validation predictions and calculated AUPR values using PROCR (Script: Run-TargetFinder-Full.sh).

To dissect features contributing to TargetFinder’s high performance on ChIA-PET pairs, we created four subsets of pairs for the GM12878 RNAPII and CTCF ChIA-PET datasets.

1. A subset with a 1:2 ratio of positives to negatives which was created by subsampling 1 positive link for each cCREs and 2 negative links for each cCRE. This was analogous to the 1:4 fixed ratio method described above.
2. A “promoter-matched” subset that only includes pairs from promoters that are in at least one positive and one negative pair. We then subsample to achieve a fixed 1:2 ratio of positives to negatives.
3. A “distance-matched subset for which we define 5 distance quantiles based on the distribution of positive pairs and sample equally from each bin maintaining a 1:2 ratio of positives to negatives.
4. A “promoter-distance-matched” subset for which we match for promoter use as described in (2) and distance as described in (3). Once again, we maintained a 1:2 ratio of positives to negatives.

#### Cross-cell-type performance

To test the cross-cell-type performance of TargetFinder, we generated core4 and core3 models for each cell type and then evaluated the models in other cell types. To prevent any overfitting, we assigned the chromCV of the test sets to match those of the training sets.

## Supplementary information

**Additional file 1: Table S1.** Accessions for relevant ENCODE cCREs and RNA-seq experiments.

**Additional file 2: Table S2.** Characteristics of BENGI datasets.

**Additional file 3: Figure S1.** Expression levels of genes in BENGI pairs. **Figure S2.** PR curves for unsupervised models. **Figure S3.** Correlation between BENGI pairs. **Figure S4.** Correlation methods perform poorly due to the ubiquity of promoters. **Figure S5.** PR curves of the supervised methods evaluated with BENGI datasets.

**Additional file 4: Table S3.** AUPRs for unsupervised methods.

**Additional file 5: Table S4.** AUPRs for supervised methods.

**Additional file 6: Table S5.** AUPRs for cross-cell type trained supervised methods.

**Additional file 7.** Review history.

## Data Availability

All processed datasets and corresponding scripts are available and version controlled at https://github.com/weng-lab/BENGI [35]. cCREs are available at the ENCODE portal (www.encodeproject.org) under the following accessions: ENCSR480YCS, ENCSR451UAK, ENCSR502KJC, ENCSR981LWT, ENCSR593JKE, ENCSR376OQT, ENCSR970YPW, ENCSR282MAC, ENCSR551OAQ, ENCSR529CSY, ENCSR117DKP, ENCSR117AJU, and ENCSR935NWB. ChIA-PET data is available at GEO under accession GSE72816. Hi-C data is available at GEO under accession GSE63525. CHi-C data is available from ArrayExpress under accession E-MTAB-2323. Cis-eQTLs from the GEUVADIS project are available at ftp://ftp.ebi.ac.uk/pub/databases/microarray/data/experiment/GEUV/E-GEUV-1/analysis_results/EUR373.gene.cis.FDR5.all.rs137.txt. GTEx single-tissue cis-eQTLs are available in GTEx_Analysis_v7_eQTL.tar.gz from https://gtexportal.org/home/datasets. crisprQTL data is available from the supplemental tables in Gasperini et al. [20]. ENCODE RNA-seq experiments are available at the ENCODE portal under the following accessions: ENCSR000COQ, ENCSR000CPR, ENCSR000AEM, ENCSR000CTQ, ENCSR000CPL, ENCSR830HIN, ENCSR860HAA.
