# Supplementary material for: A curated benchmark of enhancer-gene interactions for evaluating enhancer-target gene prediction methods
Source: Genome Biol. 2020 Jan 22;21:17. doi: 10.1186/s13059-019-1924-8 (PMC6977301; doi:10.1186/s13059-019-1924-8)
Supplement: Supplementary file 3 — Additional file 3: Figure S1. Expression levels of genes in BENGI pairs. Figure S2. PR curves for unsupervised models. Figure S3. Correlation between BENGI pairs. Figure S4. Correlation methods perform poorly due to the ubiquity of promoters. Figure S5. PR curves of the supervised methods evaluated with BENGI datasets. [file 13059_2019_1924_MOESM3_ESM.pdf]

Additional file 3: Figs S1-5

Fig S1 | Expression levels of genes in BENGI pairs. a, Violin plots depicting the distance distributions of positive cCRE-gene pairs for each BENGI dataset. The 95th percentile of each distribution is indicated by a star for each plot. **b-e** Violin plots gene expression in positive pairs for each BENGI dataset in **b,** GM12878/LCLs **c,** HeLa cells **d,** K562 cells, and **e,** CD34+ cells, HMEC, IMR-90, and NHEK cells. The median expression level (in TPM) is displayed above each violin plot. For the 3D chromatin datasets (ChIA-PET, Hi-C and CHi-C), the genes in all positive pairs and those in the in positive pairs with ambiguous pairs removed were compared, and Wilcoxon rank-sum test *p*-values are indicated. **f,** Violin plots depicting the distance distributions of cCREs-ELS to their closest gene.

Fig S2 | PR curves for unsupervised models. AUPRs for the distance (gray), average-rank (black), DNase-DNase correlation (teal), DNase-expression correlation (purple) and GeneHancer (blue) methods across each of the BENGI datasets. The top left group includes all pairs with a natural ratio. The bottom left group includes all pairs with a fixed ratio. In the top right group, ambiguous pairs are removed, and the ratio is natural. In the bottom right group, ambiguous pairs are removed, and the ratio is fixed.

Fig S3 | Correlation between BENGI pairs. Violin plots displaying the distribution of Pearson correlation coefficients computed using the DNase-DNase or DNase-expression method for positive (right, colored) and negative (left, gray) BENGI pairs. Wilcoxon rank-sum test *p*-values are indicated. The top left group includes all pairs with a natural ratio. The bottom left group includes all pairs with a fixed ratio. In the top right group, ambiguous pairs are removed, and the ratio is natural. In the bottom right group, ambiguous pairs are removed, and the ratio is fixed.

Fig S4 | Correlation methods perform poorly due to the ubiquity of promoters. a, Normalized gene expression calculated by the DNase-expression method for all genes (black) and *AKIRIN2* (green) across 112 cell types. **b,** DNase signal at EH37E0853090 and the *AKIRIN2* promoter using the DNase-DNase correlation method. Only the lymphoblastoid cell line group (purple) presents a high signal at EH37E0853090. **c-d,** Number of biosamples in the ENCODE phase 2, ENCODE phase 3, and Roadmap projects with high DNase values (Z-score > 1.64) for cCREs-TSS with promoter-like signatures (cCRE-PLS) and cCREs with enhancer-like signatures (cCREs-ELS) included in the BENGI datasets.

Fig S5 | PR curves of the supervised methods evaluated with BENGI datasets. AUPRs for the distance (gray), average-rank (black), PEP-motif (teal), TargetFinder full-model (dark purple), TargetFinder core4 (medium purple) and TargetFinder core3 (light purple) methods in each of the BENGI datasets. The top left group includes all pairs with a natural ratio. The bottom left group includes all pairs with a fixed ratio. In the top right group, ambiguous pairs are removed, and the ratio is natural. In the bottom right group, ambiguous pairs are removed, and the ratio is fixed.

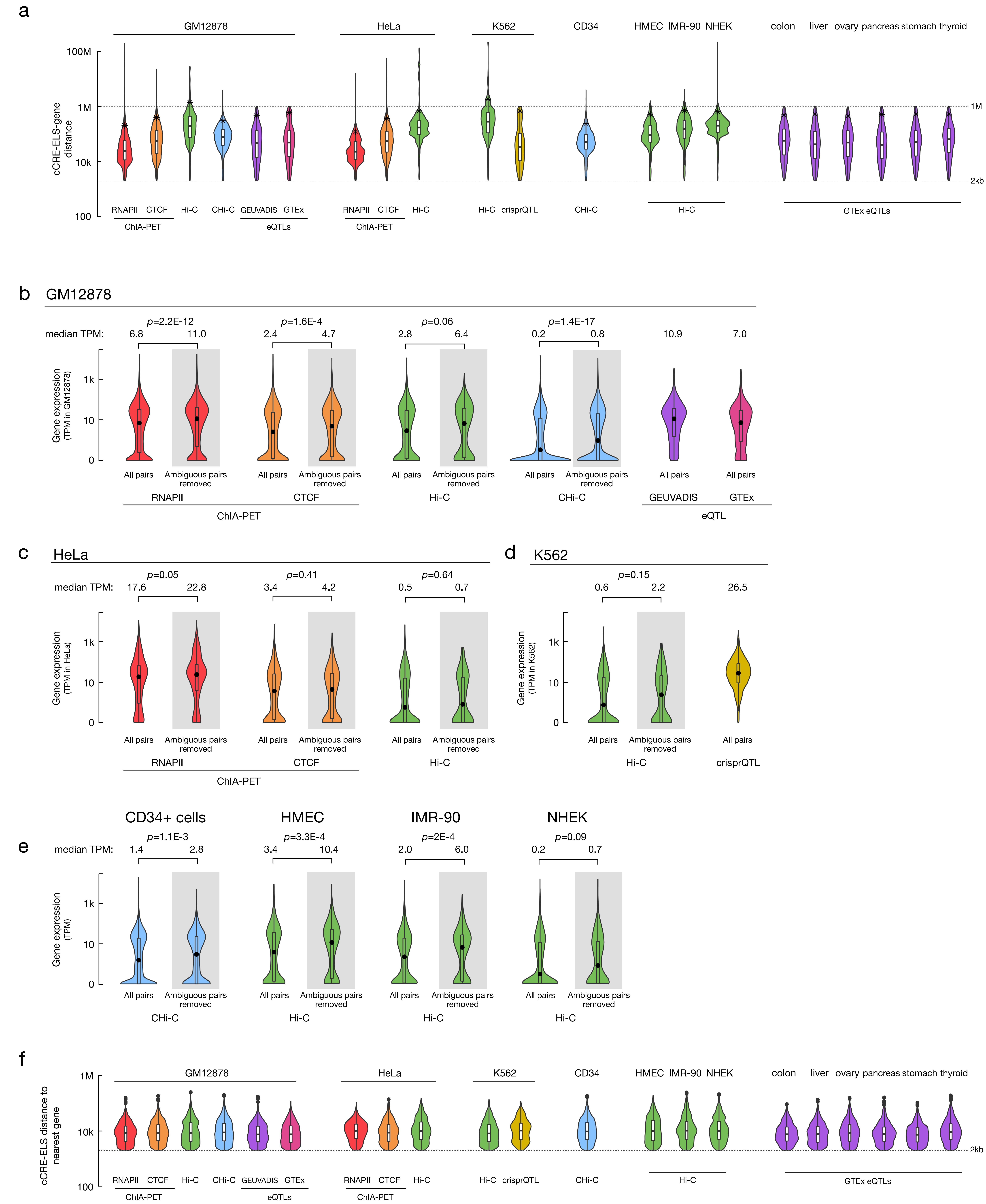
a
GM12878
HeLa
K562
CD34
HMEC IMR-90 NHEK
colon liver ovary pancreas stomach thyroid
100M
1M
10k
100
cCRE-ELS-gene distance
1M
2kb
RNAPII CTCF Hi-C CHi-C GEUVADIS GTEx
ChIA-PET
eQTLs
RNAPII CTCF Hi-C
ChIA-PET
Hi-C crisprQTL
CHi-C
Hi-C
GTEx eQTLs
b GM12878
median TPM:
p=2.2E-12
6.8 11.0
p=1.6E-4
2.4 4.7
p=0.06
2.8 6.4
p=1.4E-17
0.2 0.8
10.9 7.0
Gene expression (TPM in GM12878)
1k
10
0
All pairs
Ambiguous pairs removed
RNAPII CTCF
ChIA-PET
Hi-C
CHi-C
GEUVADIS GTEx
eQTL
c HeLa
median TPM:
p=0.05
17.6 22.8
p=0.41
3.4 4.2
p=0.64
0.5 0.7
Gene expression (TPM in HeLa)
RNAPII CTCF
ChIA-PET
Hi-C
d K562
p=0.15
0.6 2.2
26.5
Gene expression (TPM in K562)
Hi-C
crisprQTL
e
CD34+ cells
HMEC
IMR-90
NHEK
median TPM:
p=1.1E-3
1.4 2.8
p=3.3E-4
3.4 10.4
p=2E-4
2.0 6.0
p=0.09
0.2 0.7
Gene expression (TPM)
CHi-C
Hi-C
Hi-C
Hi-C
f
GM12878
HeLa
K562
CD34
HMEC IMR-90 NHEK
colon liver ovary pancreas stomach thyroid
1M
10k
100
cCRE-ELS distance to nearest gene
2kb
RNAPII CTCF Hi-C CHi-C GEUVADIS GTEx
ChIA-PET
eQTLs
RNAPII CTCF Hi-C
ChIA-PET
Hi-C crisprQTL
CHi-C
Hi-C
GTEx eQTLs

All pairs, natural ratio

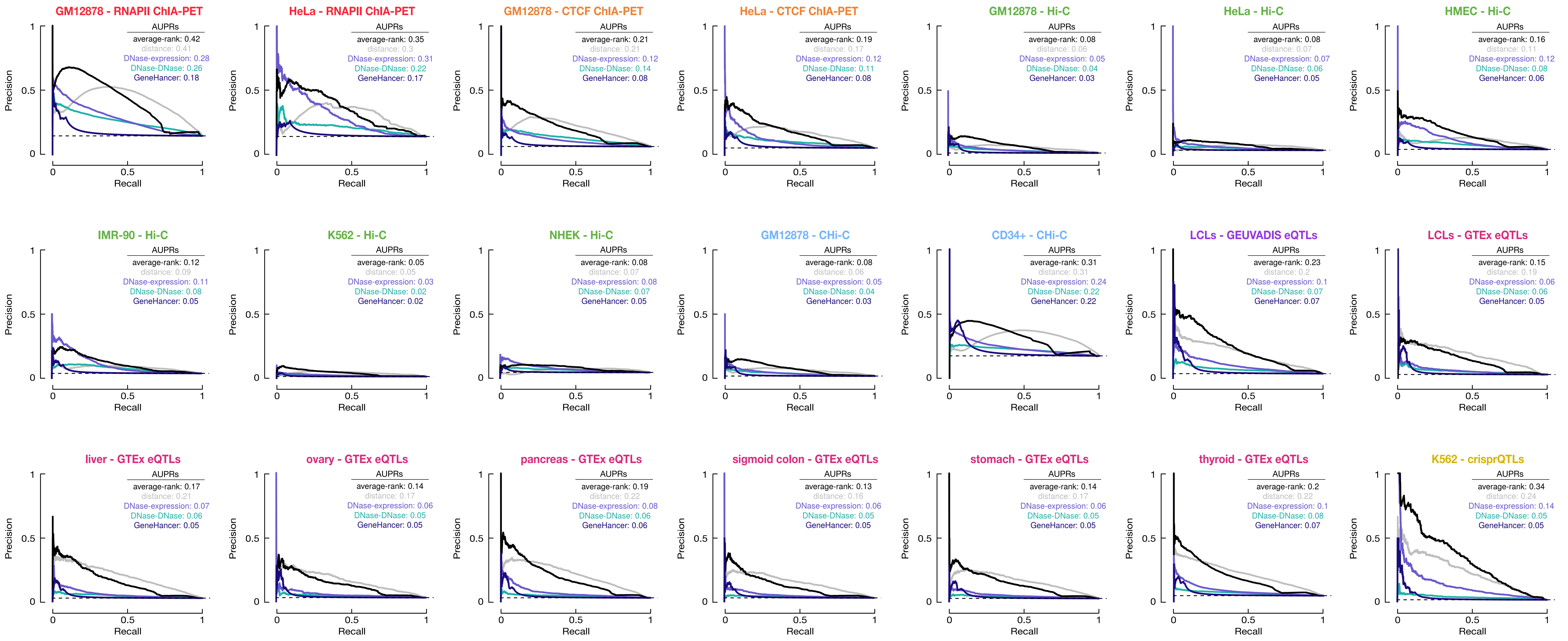

Remove ambiguous pairs, natural ratio

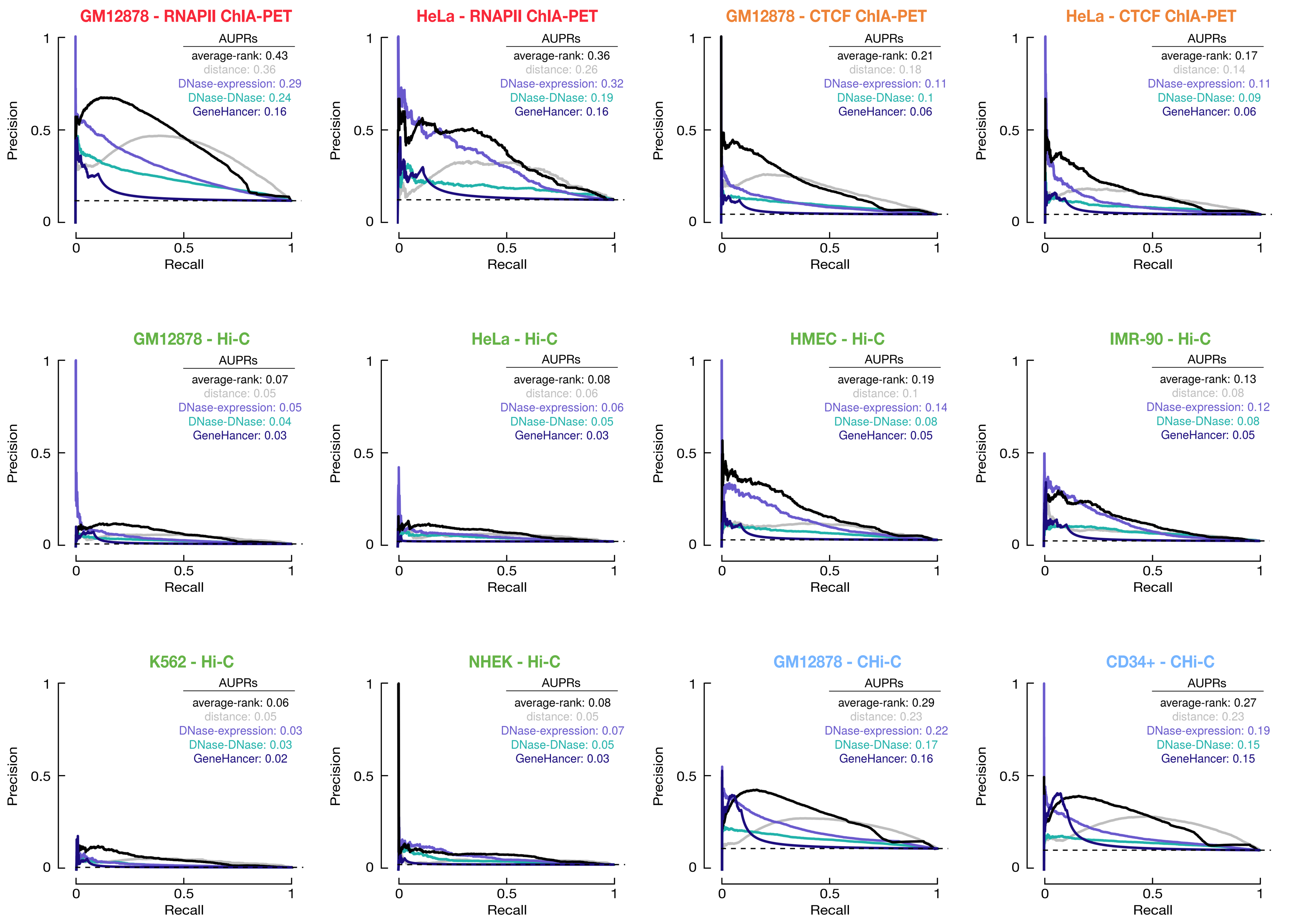

All pairs, fixed ratio

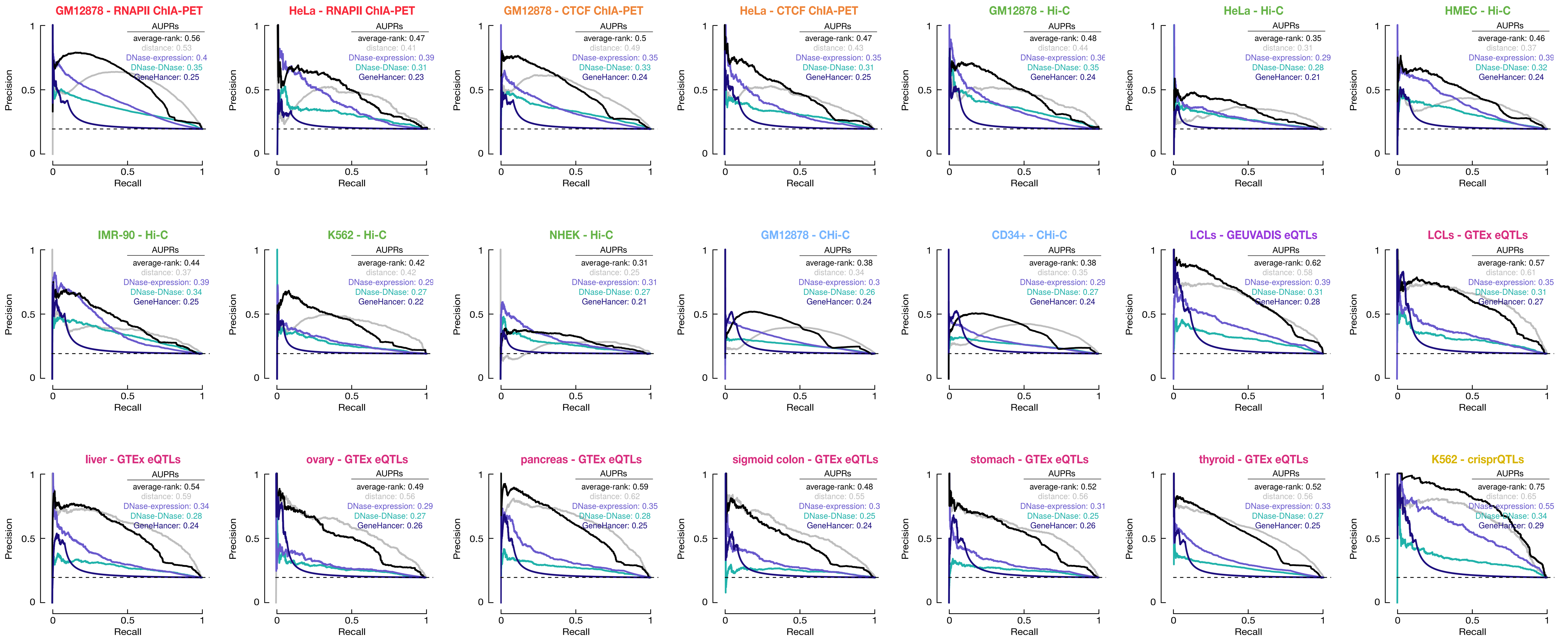

Remove ambiguous pairs, fixed ratio

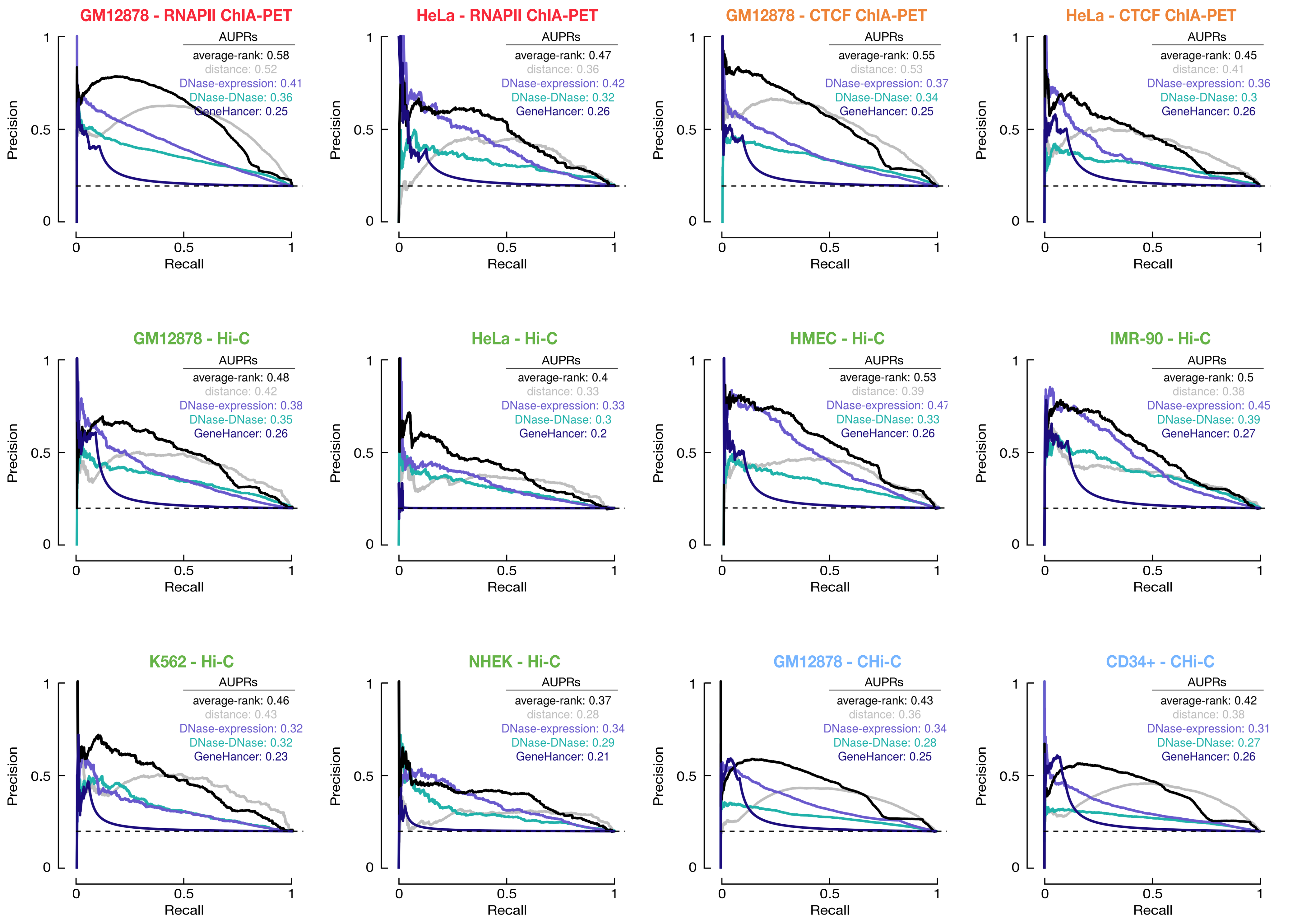

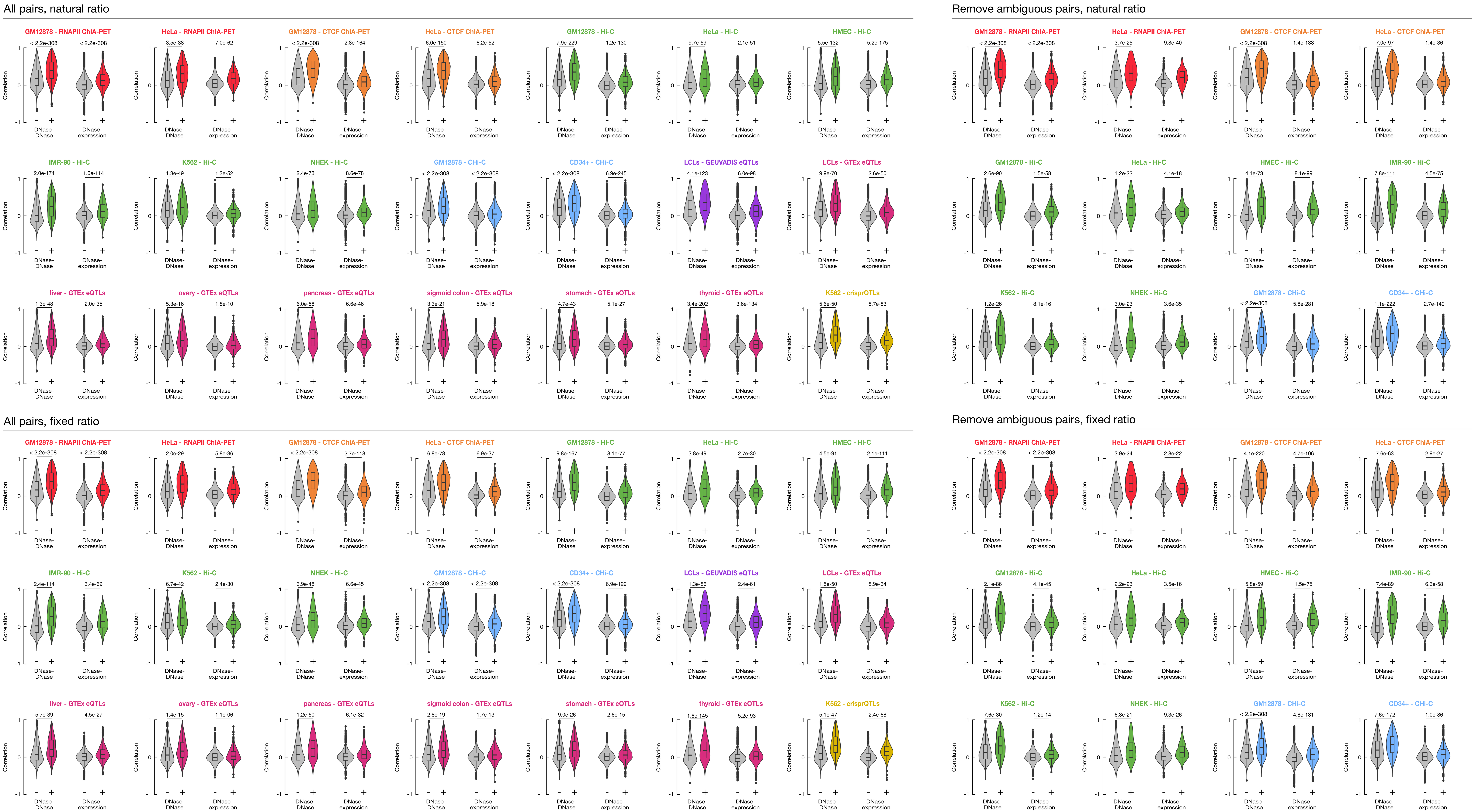
All pairs, natural ratio
GM12878 - RNAPII ChIA-PET
HeLa - RNAPII ChIA-PET
GM12878 - CTCF ChIA-PET
HeLa - CTCF ChIA-PET
GM12878 - Hi-C
HeLa - Hi-C
HMEC - Hi-C
IMR-90 - Hi-C
K562 - Hi-C
NHEK - Hi-C
GM12878 - CHi-C
CD34+ - CHi-C
LCLs - GEUVADIS eQTLs
LCLs - GTEx eQTLs
liver - GTEx eQTLs
ovary - GTEx eQTLs
pancreas - GTEx eQTLs
sigmoid colon - GTEx eQTLs
stomach - GTEx eQTLs
thyroid - GTEx eQTLs
K562 - crisprQTLs
Remove ambiguous pairs, natural ratio
All pairs, fixed ratio
Remove ambiguous pairs, fixed ratio
Correlation
DNase-DNase
DNase-expression

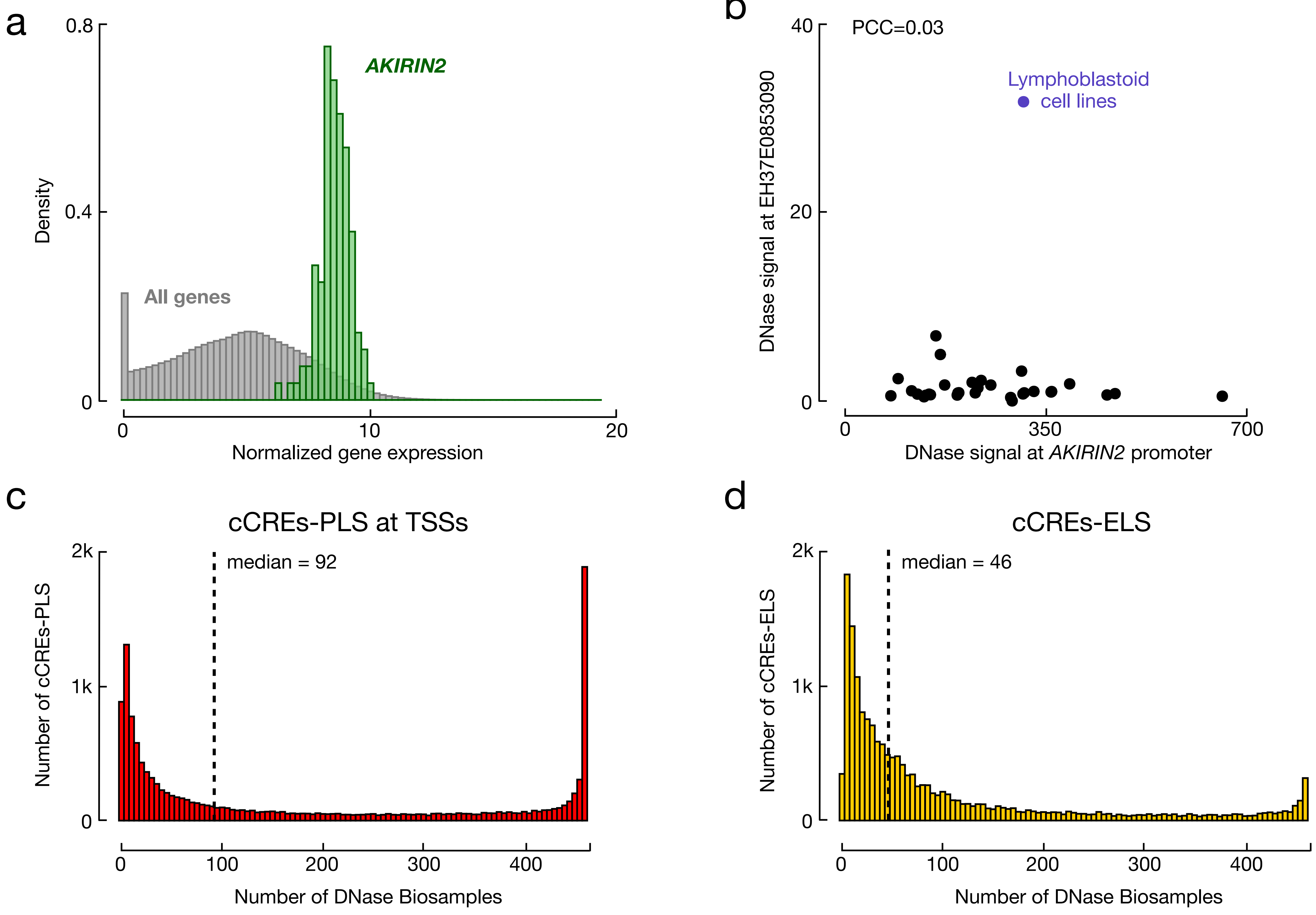
a
0.8
0.4
0
Density
AKIRIN2
All genes
0
10
20
Normalized gene expression
b
40
20
0
PCC=0.03
Lymphoblastoid
cell lines
DNase signal at EH37E0853090
0
350
700
DNase signal at AKIRIN2 promoter
c
cCREs-PLS at TSSs
2k
1k
0
median = 92
Number of cCREs-PLS
0
100
200
300
400
Number of DNase Biosamples
d
cCREs-ELS
2k
1k
0
median = 46
Number of cCREs-ELS
0
100
200
300
400
Number of DNase Biosamples

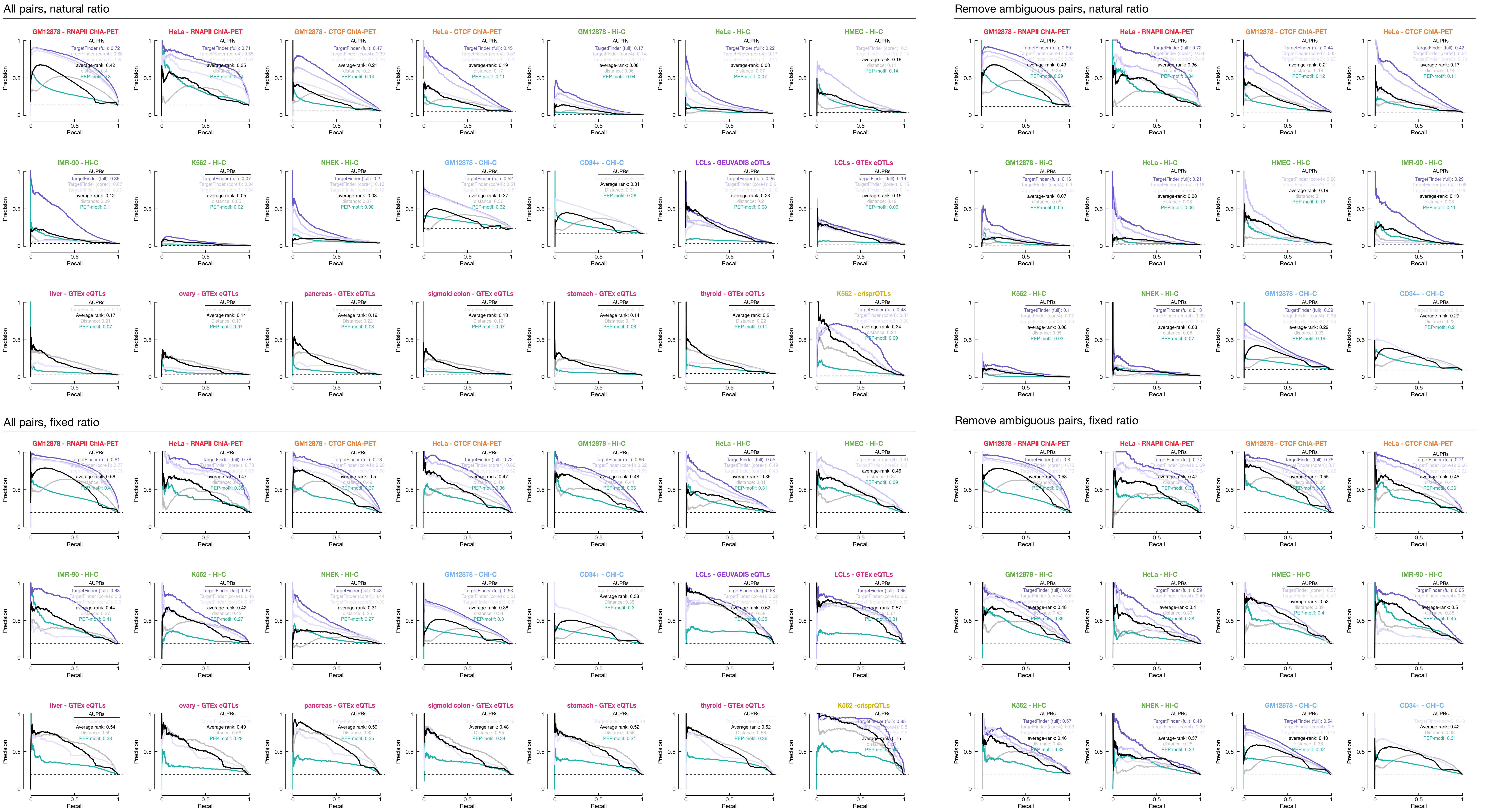
All pairs, natural ratio
GM12878 - RNAPII ChIA-PET
HeLa - RNAPII ChIA-PET
GM12878 - CTCF ChIA-PET
HeLa - CTCF ChIA-PET
GM12878 - Hi-C
HeLa - Hi-C
HMEC - Hi-C
IMR-90 - Hi-C
K562 - Hi-C
NHEK - Hi-C
GM12878 - CHi-C
CD34+ - CHi-C
LCLs - GEUVADIS eQTLs
LCLs - GTEx eQTLs
liver - GTEx eQTLs
ovary - GTEx eQTLs
pancreas - GTEx eQTLs
sigmoid colon - GTEx eQTLs
stomach - GTEx eQTLs
thyroid - GTEx eQTLs
K562 - crisprQTLs
Remove ambiguous pairs, natural ratio
All pairs, fixed ratio
Remove ambiguous pairs, fixed ratio
