# Supplementary material for: A curated benchmark of enhancer-gene interactions for evaluating enhancer-target gene prediction methods
Source: Genome Biol. 2020 Jan 22;21:17. doi: 10.1186/s13059-019-1924-8 (PMC6977301; doi:10.1186/s13059-019-1924-8)
Supplement: Supplementary file 7 — Additional file 7. Review history. [file 13059_2019_1924_MOESM7_ESM.docx]

Review History

**First round of review**

**Reviewer 1**

**Are you able to assess all statistics in the manuscript, including the appropriateness of statistical tests used?**

Yes, and I have assessed the statistics in my report.

**Comments to author:**

Mapping enhancers with their target genes is an important problem for understanding the regulatory networks in cells. This study curated a set of target genes for a group of previously predicted cis-regulatory elements, using published data from three different types of experiments. This collection of enhancer-target gene datasets will be very useful for the users in the field. They also evaluated several computational methods for predicting enhancer-gene interactions using the datasets. They used a distance-only model as the baseline for the evaluation, which provided the readers a good reference for the performance of different methods. The benchmark study was well designed and the analysis was performed appropriately.

Here are a few comments:
1) To be more comprehensive in benchmarking, other types of methods for identifying enhancer-target gene pairs may be assessed. For example, the GeneHancer, a score-based method combining enhancer-gene distance and different eipgenomics features, may be surveyed.

2) A main conclusion of the paper is that the distance-only method is actually performing better than most of the evaluated methods. More information is needed to address whether the datasets were biased against long-range enhancer-gene interactions. Figure 2a listed the distance distribution of cCRE-ELS-gene links in different datasets. It would be helpful to provide the distance distribution of selected cCRE-ELS to its closest gene for a reference. Also, besides the promoter-emphasis for ChIA-PET and CHi-C techniques mentioned in the paper, it is not mentioned that both GTEX eQTL and crisprQTL studies only tested genes within 1MB of the SNP or the enhancer of interest. Further, while the distance method worked best on the crisprQTL pairs, the distance distribution of crisprQTL pairs were not shown.

3) Although the benchmark datasets include enhancer-gene pairs across thirteen cell and tissue types, it should be noted that most of the pairs are from a few cell lines due to lack of experimental data (actually more than half of the pairs are from GM12878 or LCLs). Because the enhancer-gene interaction is often cell type-specific, the limitation should be mentioned in the discussion for the readers to decide when to use the benchmark data.

4) For the eQTL datasets, presumably only the cis-eQTLs were selected. It is not clear from the method section.

And some minor comments on figure/table formats:

1. There were two Supplementary Table 1a, one of them needs to be changed to Supplementary Table 1b.

2. Figure 2a was mentioned after Figure 2b in the text.

3. What do the two dashed lines in Figure 4a refer to?

**Reviewer 2**

**Are you able to assess all statistics in the manuscript, including the appropriateness of statistical tests used?**

Yes, and I have assessed the statistics in my report.

**Comments to author:**

Review of "A curated benchmark of enhancer-gene interactions for evaluating enhancer-target gene prediction methods," by Moore et al.  This paper develops a benchmark set of genomic interval pairs to evaluate current enhancer-promoter interaction prediction methods. The authors integrated several functional genomic data sets, including Hi-C chromatin interaction data, eQTL, and CRISPRi perturbation results. They then generated negative datasets with an enhancer-centric model and correctly applied a chromosome split cross-validation scheme to evaluate different prediction methods, with a focus on Targetfinder and PEP-motif, which utilized complex machine learning models. Given the recent identification of problems of improper training processes used by many of these methods [refs 29 & 30], it is informative to evaluate training setup effects on different data types. The authors' overall results indicated that the performance of Targetfinder didn't outperform baseline methods much, which is consistent with previous observations [ref 29 30].  This is a solid result but we have the following concern about the ChIA-PET analysis which should be addressed.

As shown in Fig 4, although Targetfinder didn't perform very well overall, it reached high accuracy in RNAPII and CTCF ChIA-PET datasets. This is likely caused by a bias in the negative dataset generation. The authors paired enhancers with unpaired promoters within a certain distance, which means that paired promoter could never exist in negative pairs. The paired and unpaired promoters are likely to be very heterogeneous for RNAPII and CTCF signal and representation in the ChIA-PET datasets, and they could presumably be easily separated by RNAPII and CTCF signals within the promoters alone. A more rigorous testing setup is to randomly sample both promoter and enhancers from distance matched and paired (EP) interactions, and test whether the models can discriminate specific interactions from non-interactions among this set.  I.e in a region with E-P-E where the left E interacts with P but right E does not, the promoter RNAIIp signal alone will not be able to distinguish which RNAPII E-P pair is positive and which is negative, and this is a better test of the model's ability to predict RNAPII CHIA-PET interactions.

**Authors Response**

**Point-by-point responses to the reviewers’ comments:**

Reviewer #1
Reviewer #1: Mapping enhancers with their target genes is an important problem for understanding the regulatory networks in cells. This study curated a set of target genes for a group of previously predicted cis-regulatory elements, using published data from three different types of experiments. This collection of enhancer-target gene datasets will be very useful for the users in the field. They also evaluated several computational methods for predicting enhancer-gene interactions using the datasets. They used a distance-only model as the baseline for the evaluation, which provided the readers a good reference for the performance of different methods. The benchmark study was well designed and the analysis was performed appropriately.

Here are a few comments:
1) To be more comprehensive in benchmarking, other types of methods for identifying enhancer-target gene pairs may be assessed. For example, the GeneHancer, a score-based method combining enhancer-gene distance and different epigenomics features, may be surveyed.

*Response: Following your advice, we added the GeneHancer method to our analysis.
We then evaluated the performance of the GeneHancer prediction model, via an integration of four types of enhancer annotations, including an earlier version of our cCREs, to generate a collection of candidate enhancers (Fishilevich et al., 2017). These candidate enhancers were then linked to genes by integrating co-expression correlations, eQTLs, CHi-C data and genomic distance. Because the authors used eQTLs and CHi-C from the same data sources as those in BENGI to build the GeneHancer model, we only evaluated the performance of the model on the ChIA-PET, Hi-C and crisprQTL pairs. While the GeneHancer predictions were better than random predictions, the differences were extremely modest (average improvement of 0.01 in AUPR). The GeneHancer predictions also had a much lower overall recall than the correlations methods (on average 8% compared to 100% and 76% for DNase-DNase and DNase-expression respectively). Even for these limited sets of predictions, GeneHancer never outperformed the DNase-expression model and only outperformed the DNase-DNase model for crisprQTLs. (Figure S3).*


2) A main conclusion of the paper is that the distance-only method is actually performing better than most of the evaluated methods. More information is needed to address whether the datasets were biased against long-range enhancer-gene interactions. Figure 2a listed the distance distribution of cCRE-ELS-gene links in different datasets.

It would be helpful to provide the distance distribution of selected cCRE-ELS to its closest gene for a reference.

*Response: We have added a panel (Figure S1f) with the distance distributions of cCREs-ELS to its closest gene for each of the BENGI datasets.*

Also, besides the promoter-emphasis for ChIA-PET and CHi-C techniques mentioned in the paper, it is not mentioned that both GTEX eQTL and crisprQTL studies only tested genes within 1MB of the SNP or the enhancer of interest.

*Response: We have updated the text to explicitly mention this bias.
Additionally, we note that the eQTL and crisprQTL interactions all have maximum distances of 1Mb (Figure S1a) because the original studies only tested SNPs within 1 Mb of each gene.*

Further, while the distance method worked best on the crisprQTL pairs, the distance distribution of crisprQTL pairs were not shown.

*Response: We have added a panel (Figure S1a) with the distance distributions of cCREs-ELS to positively linked genes for each of the BENGI datasets. Previously, our text incorrectly stated that the distance method worked best of the crisprQTL pairs—the Average-Rank method was the best performing method for these pairs. We have now corrected this mistake.*

3) Although the benchmark datasets include enhancer-gene pairs across thirteen cell and tissue types, it should be noted that most of the pairs are from a few cell lines due to lack of experimental data (actually more than half of the pairs are from GM12878 or LCLs). Because the enhancer-gene interaction is often cell type-specific, the limitation should be mentioned in the discussion for the readers to decide when to use the benchmark data.

*Response: We have updated the discussion to include this limitation in the data.
Additionally, though BENGI datasets currently span 13 biosamples, the majority of the gene-ELS pairs derived from GM12878 or LCLs because these cells have been extensively profiled. Therefore, users of the benchmark should be cognizant that not all biosamples are profiled equally. Furthermore, the remaining BENGI datasets all derive from cell lines or heterogeneous tissues, none from primary cells. We will increase the representation of primary cells in our benchmark as soon as 3D chromatin and genetic interaction data on primary cells become available.*

4) For the eQTL datasets, presumably only the cis-eQTLs were selected. It is not clear from the method section.

*Response: We updated the first paragraph of the results section and the methods section to explicitly state that we are using cis-eQTLs

For genetic interactions (cis-eQTLs) and CRISPR/dCas9 perturbations (crisprQTLs), we paired a cCRE-ELS with a gene if the cCRE overlapped the reported SNP or targeted region (Figure 1b).

We downloaded cis-eQTLs from the GEUVADIS project.

We downloaded single-tissue cis-eQTLs (GTEx_Analysis_v7_eQTL.tar.gz) from the GTEx Portal https://gtexportal.org/home/datasets.*

And some minor comments on figure/table formats:

1. There were two Supplementary Table 1a, one of them needs to be changed to Supplementary Table 1b.

*Response: We apologize for the confusion and have updated the table accordingly.*

2. Figure 2a was mentioned after Figure 2b in the text.

*Response: We apologize for the confusion and have swapped the order of the panels in Figure 2 to reflect the reference order in the text.

We obtained two clusters: one comprising the two eQTL datasets and the other comprising the four chromatin interaction datasets (Figure 2a) [...] In contrast, Hi-C identifies significantly more distant interactions than the other techniques (Figure 2b, Wilcoxon rank-sum test p-value = 1.1E-223).*

3. What do the two dashed lines in Figure 4a refer to?

*Response: We apologize for the confusion; one of the dashed lines was in error. We have updated the figure accordingly.*


Reviewer #2

Reviewer #2: Review of "A curated benchmark of enhancer-gene interactions for evaluating enhancer-target gene prediction methods," by Moore et al. This paper develops a benchmark set of genomic interval pairs to evaluate current enhancer-promoter interaction prediction methods. The authors integrated several functional genomic data sets, including Hi-C chromatin interaction data, eQTL, and CRISPRi perturbation results. They then generated negative datasets with an enhancer-centric model and correctly applied a chromosome split cross-validation scheme to evaluate different prediction methods, with a focus on Targetfinder and PEP-motif, which utilized complex machine learning models. Given the recent identification of problems of improper training processes used by many of these methods [refs 29 & 30], it is informative to evaluate training setup effects on different data types. The authors' overall results indicated that the performance of Targetfinder didn't outperform baseline methods much, which is consistent with previous observations [ref 29 30]. This is a solid result but we have the following concern about the ChIA-PET analysis which should be addressed.

As shown in Fig 4, although Targetfinder didn't perform very well overall, it reached high accuracy in RNAPII and CTCF ChIA-PET datasets. This is likely caused by a bias in the negative dataset generation. The authors paired enhancers with unpaired promoters within a certain distance, which means that paired promoter could never exist in negative pairs. The paired and unpaired promoters are likely to be very heterogeneous for RNAPII and CTCF signal and representation in the ChIA-PET datasets, and they could presumably be easily separated by RNAPII and CTCF signals within the promoters alone. A more rigorous testing setup is to randomly sample both promoter and enhancers from distance matched and paired (EP) interactions, and test whether the models can discriminate specific interactions from non-interactions among this set. I.e in a region with E-P-E where the left E interacts with P but right E does not, the promoter RNAIIp signal alone will not be able to distinguish which RNAPII E-P pair is positive and which is negative, and this is a better test of the model's ability to predict RNAPII CHIA-PET interactions.

*Response: We apologize for the confusion. Our benchmark allows a promoter to be in a positive pair with one enhancer and a negative pair with a different enhancer. We have updated the methods section to clarify this point.

Because our model is enhancer-centric, the same promoter may belong to both positive and negative sets, paired with different enhancers.

However, we do agree with you that there are some promoters exclusively in positive pairs or negative pairs. There is indeed heterogeneity in RNAPII and CTCF ChIP-seq signal between these promoter sets. This is also evident by the feature importance for the GM12878 RNAPII and CTCF TargetFinder models. Thank you for this important suggestion. Following your advice, we ran TargetFinder for the GM12878 RNAPII and CTCF ChIA-PET datasets on distance and promoter matched pairs (i.e., we only included promoters that were present in at least one positive and negative pair). We found that on these pairs, the AUPR of the full model decreased by 0.27 and 0.22 (Table S4e). Performance decreased even further for the core-4 and core 3 models (average of 0.28 and 0.32 respectively). This suggests that TargetFinder was capitalizing on the difference in the promoter ChIP-seq signal and distance distributions between positive and negative pairs. While these features are as biologically meaningful for making future predictions, they are not unique contributions from the TargetFinder algorithm. Therefore we have revised our results section and discussion section to include this important caveat:

Overall, TargetFinder's performance on the RNAPII and CTCF ChIA-PET pairs was markedly higher than its performance on other BENGI datasets. These datasets were the only two benchmarks of 3D chromatin interactions mediated by specific TFs. When we analyzed the feature-importance scores (i.e., Gini importance) from TargetFinder's GBM model, we found that RNAPII and CTCF ChIP-seq signals at promoters had the highest importance in the respective models. To further dissect the features contributed to TargetFinder's performance, we ran the algorithm on a subset of positive and negative pairs (1:2 ratio of positives to negatives) and three selections of positive and negative pairs that were matched for (i) only promoter inclusion, (ii) only distance and (iii) promoter inclusion and distance (promoter-distance, see methods). For all four subsets, the full TargetFinder still outperformed all other methods (Table S4e); however, compared to the 1:2 ratio set (average AUPR = 0.86) performance was lower for the distance-matched and promoter-matched sets (average AUPR = 0.74 and 0.69) and was the lowest for the promoter-distance matched sets (average AUPR = 0.61). We observed similar patterns with the TargetFinder core-4 and core-3 though the relative drop in performances was much larger—average decreases in AUPR of 0.25 for full model, 0.28 for core-4 model and 0.32 for core-3model. Particularly, for the core-3 CTCF ChIA-PET promoter-distance model, which does not include CTCF as a feature, we observed an AUPR of 0.43, a 0.30 reduction in AUPR compared to the 1:2 ratio pairs, and only a 0.03 improvement in AUPR over the DNase-DNase correlation method. These results suggest that differences in RNAPII/CTCF ChIP-seq signal and distance between positive and negative pairs contribute to TargetFinder's ability to successfully predict cCRE-ELS-gene pairs.

In particular, TargetFinder performed best on ChIA-PET pairs; however, the performance substantially decreased when the positive and negative pairs were matched for their distributions of RNAPII/CTCF ChIP-seq signals at promoters and cCRE-ELS–gene distances. Thus, these features are the main contributors to TargetFinder’s higher performance on ChIA-PET datasets than other BENGI datasets.*

**Second round of review**

**Reviewer 1**

The authors have addressed all my questions and I have no further comments.

**Reviewer 2**

The authors have satisfactorily addressed all our concerns in their response and we feel that the paper is now suitable for publication.
